# RNA-seq Analysis of Host and Viral Gene Expression Highlights Interaction between Varicella Zoster Virus and Keratinocyte Differentiation

**DOI:** 10.1371/journal.ppat.1003896

**Published:** 2014-01-30

**Authors:** Meleri Jones, Inga R. Dry, Dan Frampton, Manuraj Singh, Ravinder K. Kanda, Michael B. Yee, Paul Kellam, Michael Hollinshead, Paul R. Kinchington, Edel A. O'Toole, Judith Breuer

**Affiliations:** 1 Division of Infection and Immunity, University College London, London, United Kingdom; 2 Centre for Cutaneous Research, QMUL, London, United Kingdom; 3 Department of Ophthalmology and of Molecular Microbiology and Genetics, University of Pittsburgh, Pittsburgh, Pennsylvania, United States of America; 4 Virus Genomics Team, Wellcome Trust Sanger Institute, Cambridge, United Kingdom; 5 Section of Virology, Faculty of Medicine, Imperial College London, London, United Kingdom; University of Glasgow, United Kingdom

## Abstract

Varicella zoster virus (VZV) is the etiological agent of chickenpox and shingles, diseases characterized by epidermal skin blistering. Using a calcium-induced keratinocyte differentiation model we investigated the interaction between epidermal differentiation and VZV infection. RNA-seq analysis showed that VZV infection has a profound effect on differentiating keratinocytes, altering the normal process of epidermal gene expression to generate a signature that resembles patterns of gene expression seen in both heritable and acquired skin-blistering disorders. Further investigation by real-time PCR, protein analysis and electron microscopy revealed that VZV specifically reduced expression of specific suprabasal cytokeratins and desmosomal proteins, leading to disruption of epidermal structure and function. These changes were accompanied by an upregulation of kallikreins and serine proteases. Taken together VZV infection promotes blistering and desquamation of the epidermis, both of which are necessary to the viral spread and pathogenesis. At the same time, analysis of the viral transcriptome provided evidence that VZV gene expression was significantly increased following calcium treatment of keratinocytes. Using reporter viruses and immunohistochemistry we confirmed that VZV gene and protein expression in skin is linked with cellular differentiation. These studies highlight the intimate host-pathogen interaction following VZV infection of skin and provide insight into the mechanisms by which VZV remodels the epidermal environment to promote its own replication and spread.

## Author Summary

Varicella zoster virus (VZV) causes chickenpox and shingles, which are characterised by the formation of fluid-filled skin lesions. Infectious viral particles present in these lesions are critical for airborne spread to cause chickenpox in non-immune contacts and for infection of nerve ganglia via nerve endings in the skin, a pre-requisite for shingles. Several VZV proteins, although dispensable in laboratory cell-culture, are essential for VZV infection of skin, a finding thought to relate to VZV interaction with a process known as epidermal differentiation. In this, the specialised keratinocyte cells of the outer layer of skin, the epidermis, are continually shed to be replaced by differentiating keratinocytes, which migrate up from lower layers. How VZV interaction with epidermal differentiation leads to the formation of fluid-filled lesions remains unclear. We show using a keratinocyte model of epidermal differentiation that VZV infection alters epidermal differentiation, generating a specific pattern of changes in that is characteristic of blistering and skin shedding diseases. We also identified that the differentiation status of the keratinocytes influences the replication pattern of the viral gene and protein expression, with both increasing as the VZV particles traverses to the uppermost layers of the skin. The findings provide new insights into VZV-host cell interactions.

## Introduction

Replication in skin and mucosa is central to the pathogenesis of varicella zoster virus (VZV), a member of the alphaherpesvirus subfamily that causes chickenpox (varicella) upon a primary infection and shingles (herpes-zoster) following reactivation from a neuronal latent state. In both diseases, VZV replication in the epidermal layer of skin results in the formation of large polykaryocytes and the development of blisters containing infectious cell-free virus. The epidermis is a continually regenerating tissue layer that develops a stratified structure, which is maintained by keratinocytes, specialized cells which produce a network of keratin filaments anchored to intracellular junctions to provide structural support to the tissue. As keratinocytes transit from the stem-cell rich basal to the uppermost layer of the epidermis, they undergo a program of terminal differentiation. Each stratum (basal, spinous, granular, lucidum and cornified) [1] identified within the stratified epidermis is associated with established signature patterns of gene expression [2] [3]. This process is tightly regulated by homeostatic mechanisms that involve calcium gradients, microRNAs, developmental signalling pathways and proteolytic cascades [4], [5], [6], [7], [8], [9].

Although VZV infects primary cultured keratinocytes [10] little is known about the interaction between VZV replication and epidermal differentiation. Previous work has shown that VZV replication in skin differs from monolayer cultures in that certain VZV proteins, such as ORF10 and ORF11, are not required for replication in melanoma monolayer cultures but are necessary for optimal replication in foetal skin xenografts of SCID-hu mice, [11], [12]. Additionally, the live attenuated VZV vaccine, vOKA, replicates well in tissue culture but is attenuated for replication in skin but not in lymphoid or neuronal xenografts in SCID-hu mouse models [13].

In the present study we used an *in vitro* calcium induced model of epithelial differentiation [5] and analysed the transcriptome of uninfected and VZV-infected primary keratinocytes using RNA-seq. This approach identified not only the effect of VZV on keratinocytes but also the consequence of keratinocyte differentiation on VZV replication and maturation. Together our data provides intriguing new insights into host–pathogen interactions.

## Results

### VZV infection of primary keratinocytes

As keratinocytes differentiate they lose basally expressed cytokeratins (KRT5/14/15) and increase the expression of differentiation markers e.g. suprabasal cytokeratins (KRT1/10) and involucrin (IVL). The addition of calcium to primary keratinocytes in culture, mimics the calcium gradient across the epidermis and the process of epidermal differentiation [5]. To assess the effect of calcium on primary keratinocytes we measured by qPCR the change in the expression of selected keratinocyte markers known to be altered by differentiation (KRT10, KRT15 and IVL) (Figure S1) and confirmed our findings by immunoblotting for KRT10 and IVL (Figure S1). In our hands, the addition of calcium to 1.2 mM increased the expression of the suprabasal (KRT10) and granular marker (IVL) as well as reducing the expression of the basal marker (KRT15), demonstrating that we could use calcium to induce keratinocyte differentiation.

**Figure 1:**
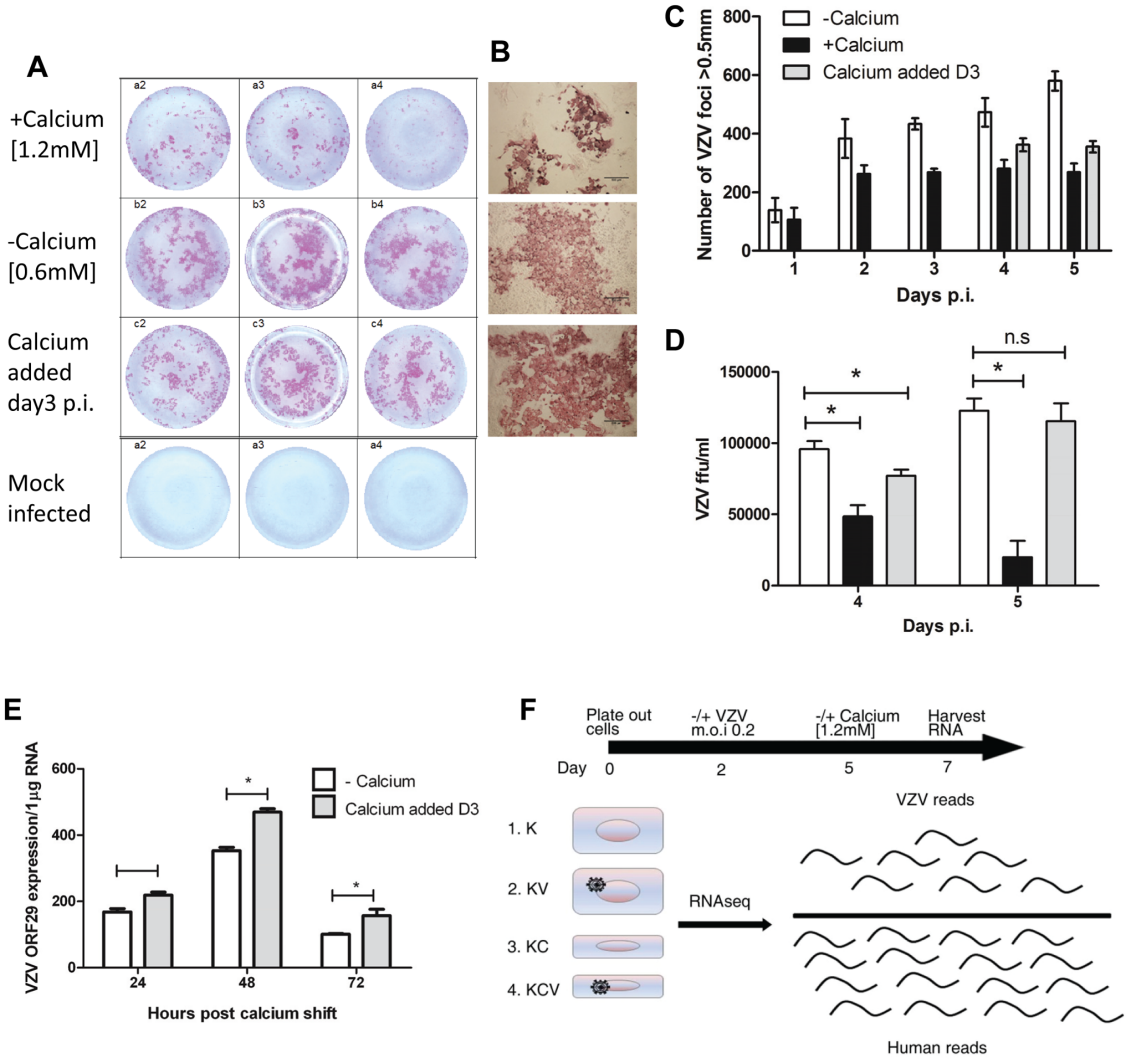
VZV infection of primary keratinocytes. Primary human keratinocyte were culture in a 24-well plate either in a low [0.6 mM] (−calcium) or high [1.2 mM] calcium (+calcium) containing media and infected with VZV at an mo.i. of 0.2. For the final condition, cells were infected with VZV as above and then at day 3 p.i. the calcium concentration was increased to 1.2 mM (calcium added Day 3). Cells were cultured for up to 5 days p.i. and fixed in 4% PFA. VZV infected cells were identified using IHC and images of the VZV colonies were captured and counted. (**A**) Representative VZV colonies at day 5 post infection in triplicate from keratinocytes grown in the low, high or switched at day 3 calcium media. (**B**) Representative images of VZV infected cells at day 5 p.i. grown in the three different conditions (scale bar = 500 µm). (**C**) The VZV foci number in primary keratinocytes was counted up to 5 days post infection and the result represented as ± standard deviation (n = 3). (**D**) VZV infected primary keratinocytes treated with trypsin and titred onto MeWo cells at days 4 and 5 p.i. VZV colonies were identified by IHC and the ffu/ml calculated and represented as ± standard deviation (n = 3) the statistical difference between the conditions was determines (p values less than 0.05 (*) are indicated. **E**) VZV infected primary human keratinocytes were processed for total RNA extraction following the calcium switch at day 3 post infection. The levels of VZV ORF29 cDNA were determined by qPCR and normalised to the housekeeping gene RN5S, experiment was carried out in triplicate, p-values of less than 0.05 (*) by Student's t-test are shown. **F**) Representation of conditions used in RNA-seq experiment, keratinocytes were infected with cell-free VZV (m.o.i of 0.2) at day 2 and either maintained in a low calcium media or changed to a high calcium media at day 5 (day 3 p.i.), RNA was harvested at day 7. Four conditions were analysed K = Keratinocytes; KV = Keratinocytes infected with VZV; KC = Keratinocytes+calcium induced differentiation; KCV = Keratinocytes infected with VZV and calcium differentiated.

The ability of VZV to infect primary human keratinocytes has previously been assessed [10]. Sexton and colleagues noted that maintaining the keratinocytes in a low calcium media prior to VZV infection resulted in a higher initial infection. We were able to infect cells grown in low calcium [0.6 mM] (−calcium) and high calcium [1.2 mM] (+calcium) media with VZV as detected by an infectious centre assay (Figure 1A–B). Over the course of a 5 day infection we observed an increase in the number of VZV foci, indicating cell to cell spread, full replication and production of infectious virus (Figure 1C). As previously observed [10] when VZV was added to cells cultured in high calcium medium, the number of foci was less and plateaued after one day, suggesting reduced replication and spread. To optimise a model with which to investigate the interaction of VZV and keratinocyte differentiation, we next examined the effect of adding calcium [1.2 mM] to cells already infected with VZV. By immunohistochemistry, the VZV plaque size was comparable to the −calcium cells (Figure 1A–B), but the number of foci counted was still less when calcium was added 3 days p.i. (Figure 1C). Primary keratinocytes in culture are known to change size [14], develop tight junctions and form clusters [15] when treated with calcium. To ensure that this did not affect the number of VZV plaques counted, the infected keratinocytes and the associated supernatants were transferred onto MeWo cells and the VZV titres calculated. VZV was not detected in the supernatants of any sample (data not shown). The VZV titre in the +calcium cells at days 4–5 p.i. was significantly less than in the −calcium cells. However, by adding the calcium to the cells 3 days after VZV infection, the VZV titres were higher than in the +calcium cells and by day 5 no significant difference was seen in the VZV titres between the −calcium cells and the cells which the calcium had been added 3 days p.i. (Figure 1D). Flow cytometry analysis of VZV keratinocytes confirmed that keratinocytes cultured in high calcium media (+calcium) express fewer VZV proteins than the cells culture in low calcium media (−calcium) but this could be increased by adding calcium at 3 days p.i. (Figure S2). Moreover, this finding was independent of whether cell-associated or cell-free VZV was used to infect cells. VZV gene expression (ORF29) was compared by real time PCR in cells grown in low calcium media (−calcium) and cells switched to a high calcium media at day 3 post VZV infection (Fig. 1E). The expression of ORF29 peaked at 48 hrs in both the comparisons with increased expression seen in the samples which had calcium added at day 3.

From the combined data above showing peak of VZV gene expression at 48 hrs following the calcium switch and the data showing increased expression of host differentiation markers at 24–48 hours following the addition of calcium (Figure S1), we determined that the optimal time point at which to examine both host and viral gene expression together was 48 hrs after calcium-induced differentiation of keratinocytes infected with VZV 3 days previously. Using this model, we compared undifferentiated (−calcium) and differentiated (calcium added at day 3 post infection) keratinocytes as well as studying the effect of the keratinocyte differentiation on the viral transcriptome (Figure 1F).

To summarise, primary human keratinocytes were plated out at day 0 and infected/mock infected with VZV at an m.o.i of 0.2 at day 2. Cells were then incubated at 34°C until day 5 before either maintaining the cultures in a low calcium media or switching to a high calcium media at day 3 p.i. Total RNA was harvested at day 7 (48 hrs after changing media and 5 days p.i.) for all four experimental conditions (K; Keratinocytes, KV; Keratinocytes and VZV, KC; Keratinocytes and Calcium and KCV; Keratinocytes, Calcium and VZV) as illustrated in Figure 1H.

The cDNA was sequenced using the Illumina RNA-seq platform. Between 15–36×10^6^ reads were generated per lane of which 4.8–12×10^6^ mapped to the human transcriptome once duplicate reads had been removed (*Homo sapiens* (release 37) reference sequence GRCh37/hg19: Table S1). Similar distributions of reads per gene were found across all samples before normalisation with no major outliers (Figure S3). The number of duplicate reads per sample varied between 21–45%, with higher levels of duplication observed in the samples from batch 3, presumably due to a PCR batch effect. However, estimated library sizes (10–32×10^6^) were independent of batch and average quality scores rose from 31 for batches 1 and 2 to 38 for batch 3, corresponding to updated Illumina reagents and protocol.

Post-normalisation, clustering transcriptome profiles by Spearman's rank correlation coefficient gave tight clusters for the calcium treated samples (KC and KCV), whilst those for the KV and K samples were more dispersed with clustering being more heavily dependent on keratinocyte batch. The exceptions were samples KV2 and KV5, which clustered tightly with the KC samples (Figure S3).

To ensure that no bias was introduced into the cDNA library, ten genes were amplified by real time PCR, and shown to have good overall correlation to RNA-seq reports when comparing the effect of virus in calcium-treated (KCV/KC) and untreated (KV/K) cells (Pearson's ρ = 0.88 and 0.75 respectively, (Figure S3).

### Overview of the human transcriptome analysis

**Figure 2:**
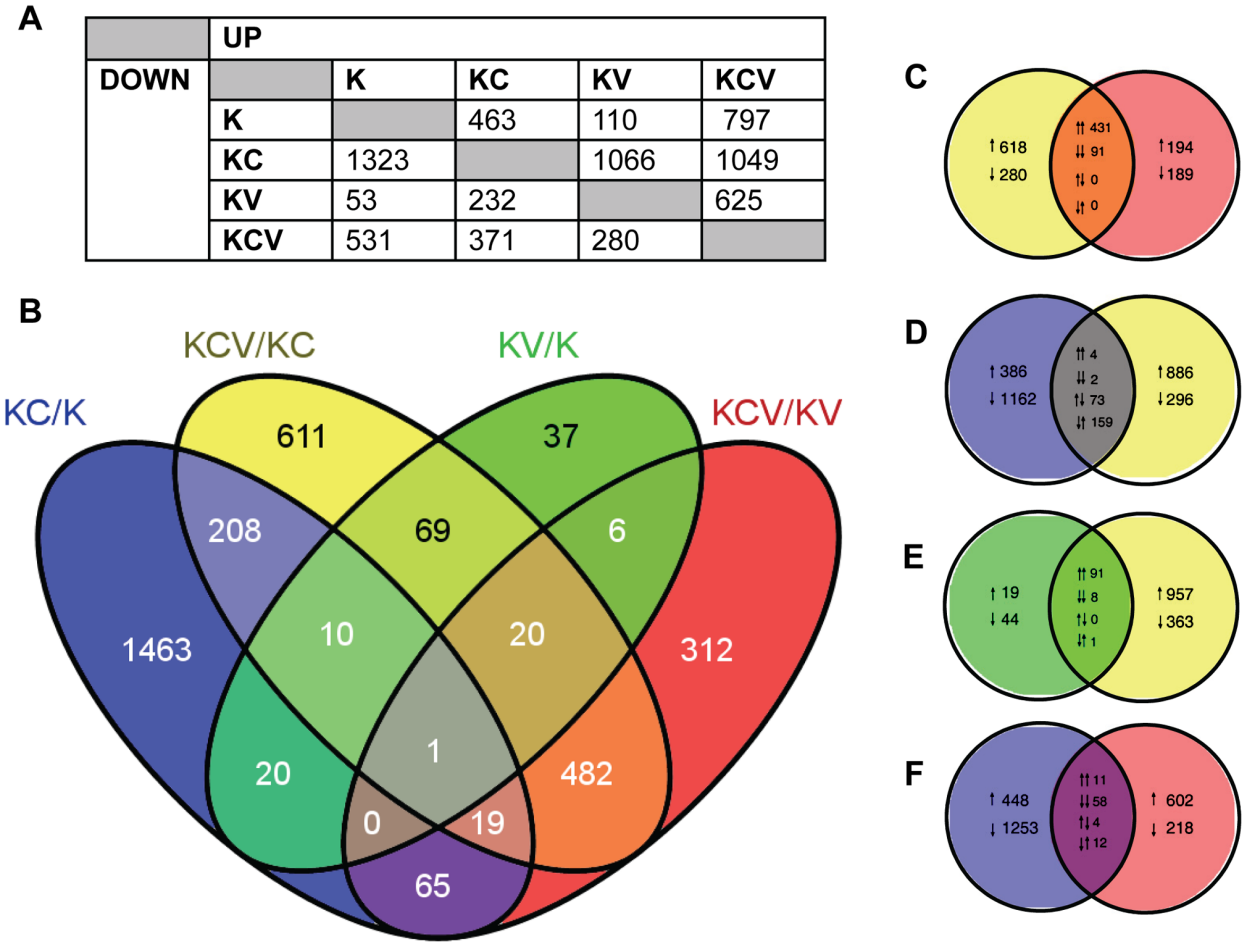
Overview of transcription data. **A**) Summary of the number of significantly up and down regulated genes (FDR<0.01) observed across all six possible comparisons of the four sample types e.g. 463 genes are up regulated in KC/K whilst 53 genes are down regulated in KV/K. **B**) Venn diagram indicating the number of significant (FDR<0.01) differentially expressed genes across four key comparisons (KCV/KC, KC/K, KV/K and KCV/KC) and the overlap between each set of genes. **C–F**) Detailed analysis between the four comparisons illustrating the overlap between up and down regulated gene lists. Pairs of arrows in the intersection refer to the direction of fold change in the comparisons on the left and right hand sides respectively. Comparisons are shown for: **C**) KCV/KV and KCV/KV, **D**) KC/K and KCV/KC, **E**) KV/K and KCV/KC and F) KC/K and KCV/KV genes. For these four Venn diagrams (e.g comparison X vs. comparison Y), those genes that are up or down-regulated in X but not significantly altered in Y are shown on the left-hand side with single arrows denoting the direction of fold change. The converse is shown on the right-hand side (i.e. genes that are differentially expressed in Y but not in X), again with up or down arrows denoting the direction of fold change. The overlaps themselves show the 4 possible options: up in both X and Y, down in both X and Y, up in X and down in Y, or down in X and up in Y. These are denoted by pairs of arrows, with the left-hand arrow referring to the direction of fold change in X and the right-hand arrow denoting the direction of fold change in Y e.g. in KC/K vs. KCV/KC (**D**), 4 genes are up regulated in both comparisons, 2 are down regulated in both comparisons, 73 are up regulated in KC/K but down regulated in KCV/KC whilst 159 are down regulated in KC/K but up regulated in KCV/KC.

A negative binomial generalised log-linear model was fitted to the TMM-normalised read counts of 17463 human genes with reads above a threshold of 1 Count Per Million (CPM) in at least 3 samples. From this, likelihood-ratio tests were performed, identifying 3863 differentially expressed genes across 6 comparisons of interest (KC/K, KV/K, KCV/KC, KCV/KV, KCV/K and KV/KC), (Figure 2A–B).

The greatest degree of differential expression was seen by the addition of calcium to uninfected keratinocytes (KC/K). A total of 1786 genes were altered (463 genes up-regulated, 1323 genes down-regulated), the pattern of which was consistent with the induction of keratinocyte differentiation with a decrease in the expression of basally expressed cytokeratins and an increase in the expression of most but not all of genes expressed in both the suprabasal and granular layers (Figure S4) and in keeping with the known limitations of the keratinocyte calcium-switch model, we did not see changes in genes that are expressed in the cornified layer. In contrast, relatively few genes were significantly altered by viral infection alone (KV/K, 110 upregulated, 53 downregulated). Although approximately the same viral titres were achieved in both conditions, (Figure 1D) significantly more genes were differentially expressed in the KCV/KC comparison (1049 upregulated, 371 downregulated). Of the 107 genes differentially expressed following viral infection in both differentiated and undifferentiated keratinocytes (KCV/KC and KV/K), 99 were found to be upregulated and 8 downregulated in both comparisons (Figure 2E), suggesting a common role for these genes in host response to viral infection regardless of differentiation. As we could not achieve 100% infection in either the KCV or KV samples (Figure 1), we must consider that the uninfected bystander cells in these samples could also contribute to the overall gene expression changes observed.

Only 31 genes were found to be differentially expressed in both virally infected (KV/K) and calcium treated (KC/K) conditions (19% KV/K, 2% KC/K) (Figure 2B), strongly suggesting that viral infection does not drive differentiation. A similar analysis of the KCV/KC and KC/K comparisons identified 238 genes as differentially expressed in both contrasts (17% KCV/KC, 13% KC/K). However of these genes, 159 were upregulated in the KCV/KC comparison but downregulated in KC/K, whilst 73 were downregulated in the KCV/KC but upregulated in the KC/K comparison. The direction of changes was the same for only 3% (6/238) of those genes differentially expressed in both conditions. This finding further supports the contention that VZV infection does not drive differentiation and raises the possibility that VZV may in fact interfere with or hinder it. Some of the effects seen in the VZV infected samples that were calcium switched (KCV) were also apparent in VZV infected samples that were untreated (KV). However the differences were not due solely to the effect of calcium on keratinocytes. If this were the case, we ought to see good agreement between those genes differentially expressed in both KCV/KV and KC/K. That this overlap is relatively small (85 genes: 5% total KC/K; 9% total KCV/KV) is in keeping with the observation that the KCV samples were transcriptionally distinct from both KC and K samples and reinforces the notion of an interaction between VZV infection and the process of keratinocyte differentiation.

In addition, there is only a relatively small overlap between genes differentially expressed following addition of calcium and those differentially expressed upon addition of calcium and viral infection (215 genes, 12% KC/K, 16% KCV/K). Of these genes, the direction of fold change is identical for almost all (91%, 50 genes upregulated in both comparisons, 145 genes downregulated in both comparisons) suggesting the KCV samples possess a more differentiated keratinocyte phenotype than the K samples. However, the vast majority of genes found to be differentially expressed in the KCV/K comparison (1113 genes, 84% KCV/K) are not significantly up- or downregulated by the addition of calcium alone, further supporting the notion that changes in gene expression between the K and KCV samples are not solely a consequence of keratinocyte differentiation.

**Figure 3:**
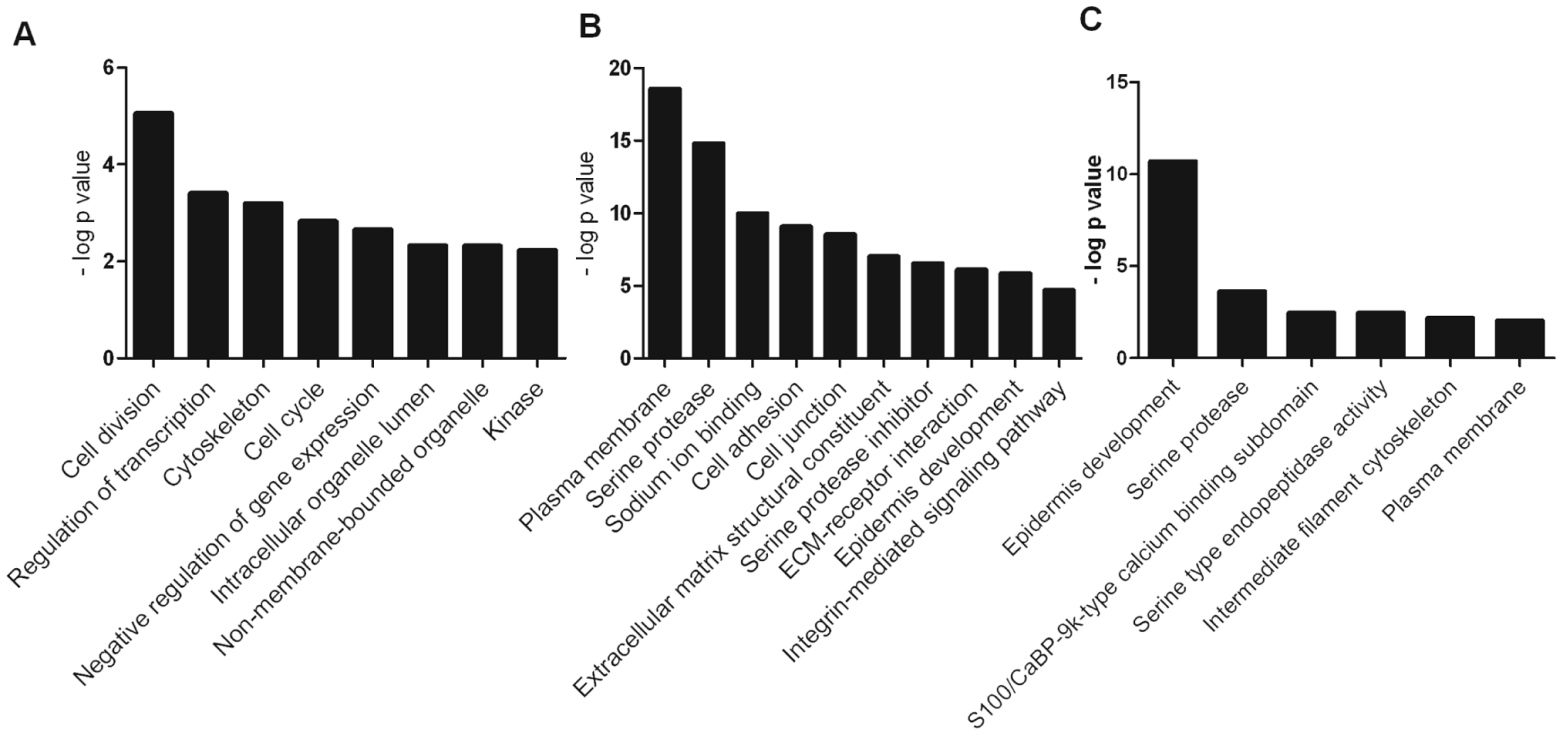
Representative functional groups enriched in differentially expressed gene lists. The genes which were differentially expressed in the (**A**) KC/K (**B**) KCV/KC and (**C**) KV/K comparisons were analysed using the functional annotation tool in DAVID. Enriched functional groups (Benjamini-Hochberg adjusted P-value<0.05) were identified for each comparison and groups representative of overall enrichment results for each comparison are shown.

Gene set enrichment analysis using the online DAVID functional annotation resource [16] identified significantly enriched functional groups (P_BH_<0.05) altered by either calcium (KC/K) (Figure 3A), by VZV infection of calcium differentiated cells (KCV/KC) (Figure 3B) or by VZV infection of undifferentiated cells (KV/K) (Figure 3C). Genes upregulated by calcium treatment of uninfected keratinocytes (KC/K) showed enrichment for several functional groups including cell cycle (GO:0007049) and cell division (SwissProt PIR keyword) whilst those that were downregulated in this comparison included regulation of transcription (GO:0045449) and negative regulation of gene expression (GO:0010629). The categories identified in the KCV/KC comparison were more varied with enrichment in the upregulated genes for several functional groups including cell junction genes (GO:0030054), the ECM-receptor interaction pathway (KEGG hsa04512) and serine protease inhibitors (SwissProt keyword). Enrichment was also observed for additional groups such as cell adhesion (GO:0007155), epidermis development (GO:0008544), serine proteases (SwissProt PIR keyword) and the integrin-mediated signaling pathway (GO:0007229). Although relatively few genes were significantly altered by viral infection alone (KV/K), the upregulated genes showed functional enrichment for epidermis development (GO:0008544) and serine-type endopeptidase activity (GO:0004252) as observed for KCV/KC. Although several interferon-stimulated genes were significantly changed after VZV infection, the corresponding GO terms (GO:0071357) were not identified as being significantly enriched in either of the VZV infected conditions (KCV or KV).

### VZV replication dysregulates cytokeratin expression

**Figure 4:**
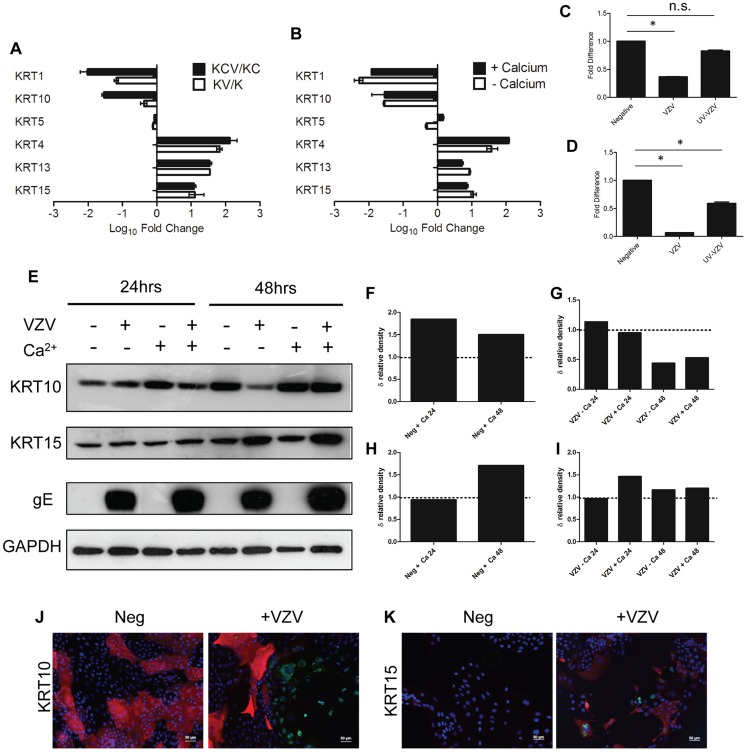
VZV infection dysregulates the expression of epidermal cytokeratins. **A**) Average fold change for KCV/KC and KV/K comparisons for epithelial cytokeratins from the RNA-seq data, showing upregulation of basal (*KRT15*) and mucosal *(KRT4/13)* and downregulation of the suprabasal cytokeratins *(KRT 1/10)*. VZV infected and mock-infected primary human keratinocytes were processed for total RNA and protein extraction. At day 5, 48 hrs after the addition of calcium, the levels of epidermal cytokeratins were determined by qPCR and normalised to the housekeeping gene RN5S (**B**). The fold change between the uninfected and VZV infected samples with and without the addition of calcium was determined and plotted ± stdev. A representative graph from 4 individual experiments is shown and the dataset is comparable to the RNA-seq analysis for all the KRT genes tested. The VZV inoculum was UV irradiated prior to infection and the qPCR was repeated for (**C**) *KRT1* and (**D**) *KRT10*. Fold change was calculated for the VZV and UV-VZV relative to the mock control for both genes, p-values<0.05 are shown (*). VZV infection again downregulated both KRT1 and KRT10, but the downregulation was not observed in the UV-treated VZV for KRT1 and was partially restored for KRT10. Quantification of VZV ORF68 was used to confirm absence of viral transcripts in UV-VZV treated cDNA (data not shown). **E**) Protein extracts were analysed by immunoblotting for KRT10 and −15 at 24 and 48 hrs after the calcium switch. The VZV infected cells are denoted by the presence of the late viral protein gE and GAPDH was used as a loading control. Change in density of KRT10 (**F**) and KRT15 (**H**) expression after the addition of calcium relative to the no calcium control were calculated using imageJ from figure 4E after VZV infection (**D**) *KRT1*, (**E**) *KRT10* and (**F**) *KRT15*. The change in relative density of KRT10 (**G**) and KRT15 (**I**) by VZV infection was calculated against the mock infected control at each timepoint. VZV infection reduced KRT10 expression and increased KRT15 expression after 48 hrs regardless of the addition of calcium. **J–K**) Immunofluorescent staining of calcium treated primary keratinocytes from uninfected and VZV samples. *KRT10* (red) was downregulated and *KRT15* (red) was upregulated in the presence of VZV expression as reported by GFP-ORF23 (green). DAPI is shown in blue. Scale bar 50 µm. The data are representative of three individual experiments.

A hallmark of epidermal differentiation are the changes that occur in cytokeratin (KRT) expression [6]. Basal cytokeratins (KRT5/14/15) are lost and suprabasal cytokeratins (KRT1/10) are gained as the keratinocytes differentiate and migrate outwards. Gene-annotation enrichment analysis using the online DAVID database of both the KCV/KC and KV/K deduced that infection with VZV profoundly affected the development of the epidermis (Figure 3B–C). Specifically, we show here that VZV alters the expression of several epidermal cytokeratins, regardless of the differentiation status of the keratinocyte (Figure S5, Table S2). The effect of VZV infection on the epithelial cytokeratins was independently verified by qPCR and the fold change calculated for a direct comparison to the RNA-seq data (Figure 4A–B). A good concordance was seen between the methods. VZV infection increased the expression of KRT15, a stem cell marker located in the hair follicle isthmus in both the KCV/KC and KV/K comparisons, although other stem cell markers (ITGB1, CD34 and CD200) (data not shown) and other basal layer cytokeratins (KRT5/14) were not altered. At the same time, VZV either down-regulated or prevented up regulation of the suprabasally expressed cytokeratin heterodimers KRT1 and KRT10, which are the major cytokeratins associated with keratinocyte differentiation [17] in both the KCV/KC and KV/K comparisons. EM images of differentiated keratinocytes infected with VZV show an abundance of cytokeratin structures (Figure S5). This finding may be related to the upregulation of KRT4/13 (131-fold and 34-fold respectively for the KCV/KC comparison, figure 4A), KRT4/13 are normally present as heterodimers in the suprabasal layers of mucosal but not stratified epithelium [18], and are thought to function like KRT1/10 to maintain cellular architecture. Upregulation of KRT4/13 may therefore have compensated structurally for the reduced expression of KRT1/10. To test whether VZV replication was responsible for the reduction of KRT1/10 expression, the qPCR experiment was repeated with UV-inactivated VZV (Figure 4C–D). We again saw a downregulation of the KRT1 and KRT10 gene expression, but these changes were partially abolished by pre-treatment of the viral inoculum by UV irradiation. We also determined the effect of VZV infection on KRT10 and KRT15 protein expression by western blotting. KRT10 expression was increased by the addition of calcium by 24 hrs. However, in the VZV infected cells the KRT10 levels were reduced and this reduction was more pronounced by 48 hrs p.i. in the undifferentiated cells, confirming that the virus downregulates KRT10 regardless of the differentiation status of the cell. KRT15 expression was upregulated by VZV infection at 48 hrs p.i. and again this effect was not dependent on the addition of calcium. Further examination of the VZV infected keratinocytes by immunofluorescence confirmed that not all cells were infected at day 5 p.i. but, KRT10 expression was absent in the ORF23GFP expressing cells. However, KRT15 expression was widespread and not necessarily confined to the VZV infected cells, confirming that not all the gene changes seen in our transcriptome data was a direct result of VZV infected cells and that changes in the bystander cells also contribute to the changes seen (Figure 4F).

**Figure 5:**
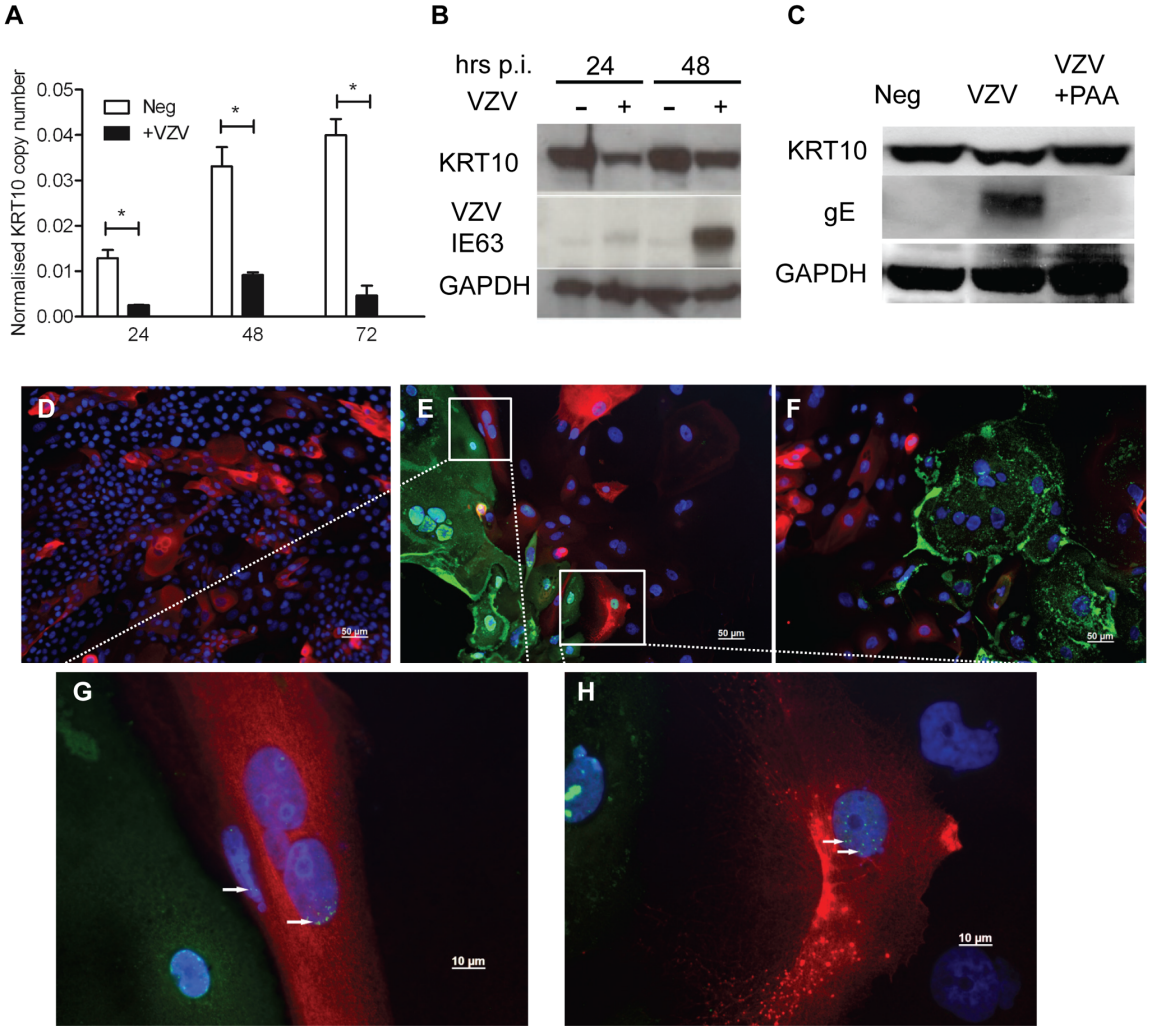
Confirming the downregulation of KRT10 by VZV in a keratinocyte cell line. nTERTs were infected with an m.o.i of 0.2. and processed for RNA and protein extraction. **A**) KRT10 gene expression is reduced by VZV infection up to 72 hrs p.i. as measured by real time PCR and normalised to RN5S. KRT10 expression increases in the mock infected cells as they become more confluent. Experiment carried out in quadruplicates and p-values calculated by Student's t-test p<0.05 (*). **B**) KRT10 protein levels are reduced at 24 and 48 hrs in VZV infected cells which are shown by the presence of the ORF63 protein, GAPDH was used as a loading control. **C**) Pre-treatment of cells with PAA, which inhibits VZV DNA polymerase and viral replication prevents the reduction of KRT10 expression measured by western blot. Immunofluorescence showing downregulation of KRT10 in VZV infected nTERTs, KRT10 staining (red) is abundant in the no virus control (**D**) whereas in cells positive for IE62 (**E**) or gE (**F**) (both green), KRT10 protein expression is absent, DAPI is shown in blue, scale bar = 50 µm. **G–H**) Higher magnification of (**E**), show that early in VZV infection, when IE62 expression (green and indicated by white arrows) is confined to the nuclei, KRT10 expression is still present in the VZV infected cells, scale bar = 10 µm.

The transcriptome data was confirmed for KRT10 using a keratinocyte cell line, nTERTs. As with the primary keratinocytes, we were unable to achieve100% infection of the nTERTs even at an m.o.i. = 2 (as measured in MeWo cells) after 5 days and it was easier to establish VZV infection in sparsely plated nTERTs (data not shown). As the nTERTs become more densely populated, the expression of KRT10 increased over the course of the experiment as measured by real time PCR. However infection of these cells with VZV significantly reduced KRT10 gene expression (Figure 5A). The downregulation of KRT10 by VZV in nTERTs was also observed at the protein level (Figure 5B). At both 24 and 48 hrs post infection the expression of KRT10 was reduced in VZV infected nTERTs compared to the mock infected controls and pre-treatment of the viral inoculum with PAA, which inhibits VZV DNA polymerase and viral replication restored KRT10 expression (Figure 5C). To assess the expression of KRT10 in the presence of VZV infection, the cells were examined by immunofluorescence. The nTERTs are a heterogenous cell population and not all the cells express KRT10, as shown in the mock infected control (Figure 5D). However, KRT10 expression in VZV infected cells was diminished, particularly in infected cells expressing the late protein (gE) (Figure 5F). Closer examination of the VZV infected showed that a number of cells where the expression of ORF62 was confined to the nuclei, which is indicative of an early VZV infection [19], still expressed KRT10 (Figure 5G–H), which in addition to the UV-treated virus and PAA data indicates that the effect of VZV on KRT10 is dependent on viral replication.

**Figure 6:**
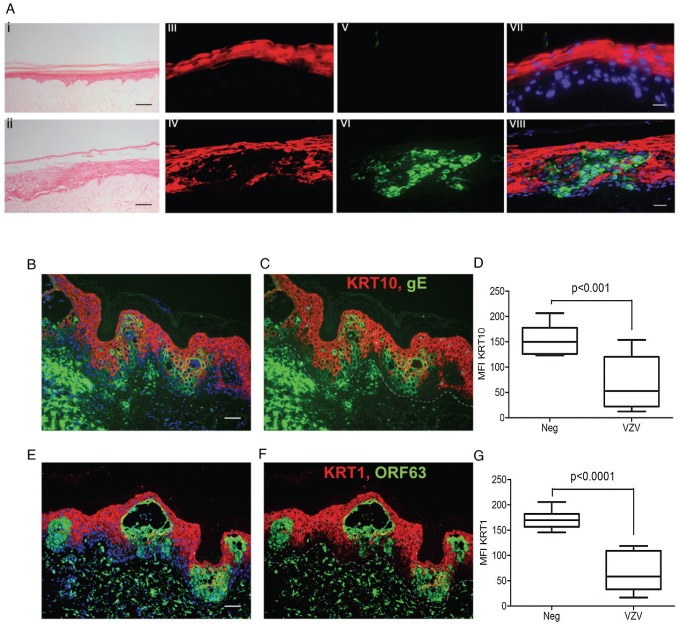
VZV downregulated *KRT1/10* expression in the epidermis. **A**) H&E of a cross-section of VZV infected primary organotypic rafts to show swollen cells within the intact epidermis, typical of early VZV lesion formation which are not seen in the mock infected control, scale bar 200 µm. *KRT10* (red) expression is suprabasal in the mock control (iii) but reduced in VZV infected pocket (vi) as indicated by VZV gE (green). Panels vii and viii represent the merged images with DAPI stained nuclei. Scale bars 25 µm. (**B–G**) Immunofluorescent staining of human varicella skin biopsies, showing *KRT10* (red, top panels) and *KRT1* (red, bottom panels) expression in the spinous layer. Dashed line represents the dermis/epidermis junction. The loss of both KRTs are seen in VZV positive areas (green), scale bars 50 µm. **D** and **G** represent the mean fluorescent intensity (MFI) of *KRT10* (**D**) and *KRT1* (**G**) from infected and uninfected cells from the spinous layer. Ten infected (green) and ten uninfected (not green) cells were taken from the spinous layer and the intensity of the KRT10 fluorescence (red) measured from each using imageJ. The mean intensity of KRT10 was then measured ± standard deviation and the associated p values calculated by a Student's t-test.

To determine whether the VZV associated downregulation of KRT10 seen in VZV infected keratinocyte monolayers occurred in more physiologically representative skin models, keratinocyte organotypic rafts were infected with VZV. Organotypic raft cultures are an *in vitro* system that recapitulates epithelial differentiation and have previously been used to study VZV replication in keratinocytes [20]. H&E staining revealed intact but swollen cells in the VZV infected raft, which are typically seen in early VZV skin lesions (Figure 6A). KRT10 expression was confined to a continuous layer in the suprabasal region of the mock-infected raft (Figure 6A vii) but disrupted in the VZV infected raft (Figure 6A viii), with no expression of KRT10 seen in the VZV infected pocket (indicated by VZV gE expression). These findings were also confirmed in skin biopsy samples from VZV cases (Figure 6B–G). As previously observed, KRT10 expression occurred in the suprabasal layers of the epidermis and gross examination suggested downregulation of KRT10 expression restricted to VZV antigen positive infected areas (Figure 6B–C). KRT10 mean intensity was compared in uninfected and VZV infected cells within the suprabasal layer of the epidermis. Ten VZV positive and ten VZV negative cells were selected within the suprabasal layer of the epidermis and the fluorescence of the KRT10 staining (red) was measured using ImageJ and found to be significantly less in the cells staining positive for VZV gE (Figure 6D). As KRT10 forms heterodimers with KRT1 within the suprabasal layer of the epidermis [18], we also examined KRT1 staining in a biopsy sample and carried out the same analysis of the fluorescence intensity in VZV positive and negative cells within the suprabasal layer (Figure 6E–F). In agreement with the KRT10 result, KRT1 expression was also substantially reduced in VZV infected cells (Figure 6G).

### VZV dysregulates desmosomes

**Figure 7:**
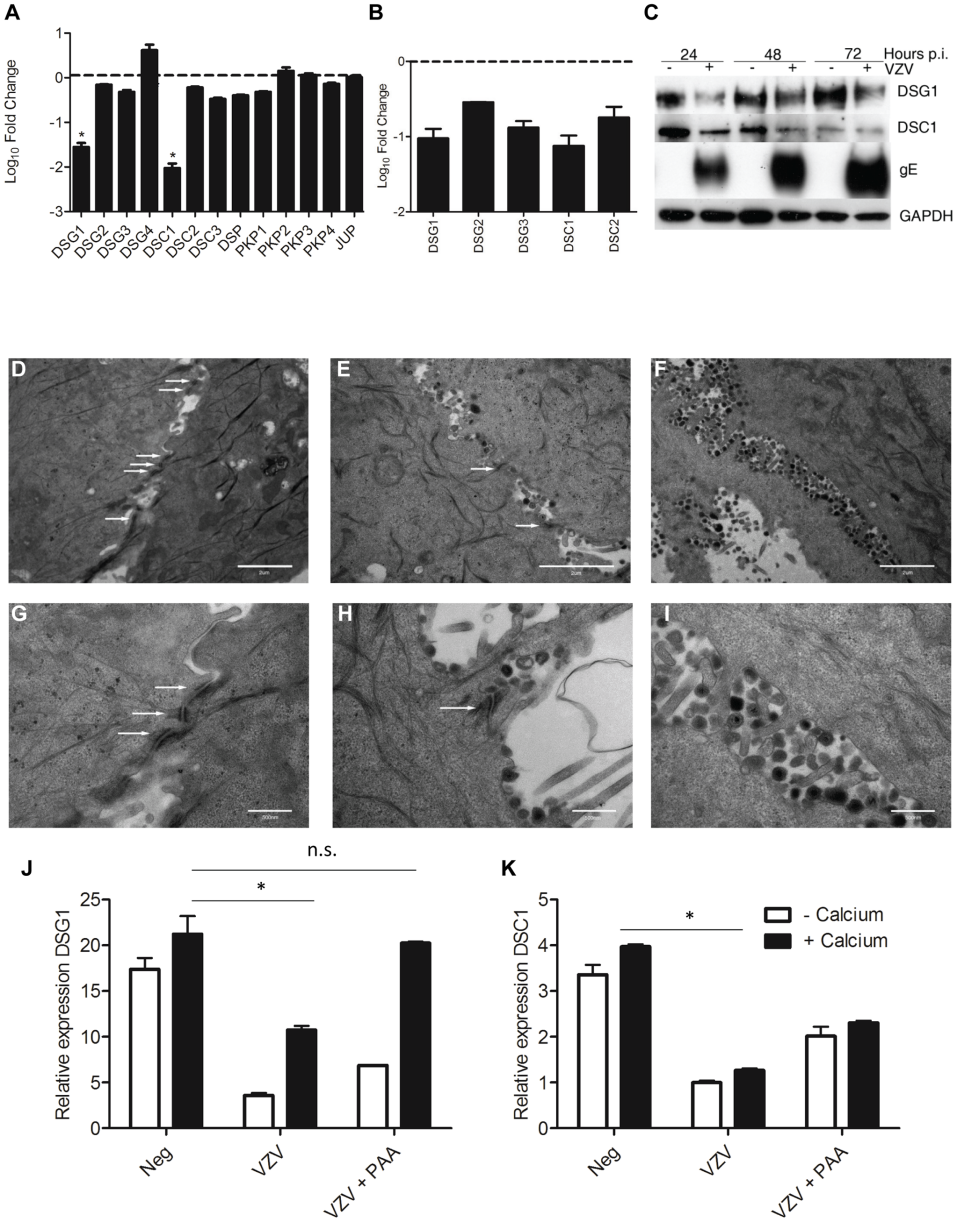
VZV infection affects the integrity of the epidermis by disrupting the desmosomal junctions. From the RNA-seq dataset the average fold change (KCV/KC) show that genes associated with the desmosomes (**A**) were significantly downregulated (*p<0.01) in differentiated keratinocytes after VZV infection. **B**) VZV infected and mock-infected primary human keratinocytes were processed for total RNA and protein extraction. At day 5 p.i., 48 hrs after the addition of calcium, the levels of the desmosomal genes were determined by qPCR and normalised to the housekeeping gene RN5S (**B**). The fold change between the uninfected and VZV infected samples was determined and plotted ± stdev. **C**) Immune blotting to confirm decreased *DSG1* and *DSC1* expression in VZV infected keratinocytes. Electron microscopy images of keratinocytes that were (**D+G**) uninfected, (**E+H**) early in VZV infection as shown by the presence of viral envelopes but not complete virions, and **F+I**) Late in VZV infection, where intact virions are easily detectable. White arrows denote the desmosomal junctions, which are absent in the last panels, scale bar **D–F** = 2 µm and **G–I** = 500 nm. Pre-treatment of keratinocytes with PAA, which inhibits VZV DNA polymerase and viral replication prevents the reduction of DSG1 (**J**) and partially restores DSC1 expression (not significant) (**K**) expression measured by qPCR, n = 3 *p<0.05 by Student's t-test.

KRT1/10 bundles provide the cytoskeletal structure in the suprabasal layers of the epidermis by interacting with desmosomal proteins [21]. Detailed analysis of the intracellular structures (GO:0030054 and GO:0007155, data not shown) indicate that VZV infection altered and downregulated the expression of desmosomal components (Figure 7A, Table S3), particularly Desmoglein1 (DSG1) and Desmocollin1 (DSC1). Both of these genes are intrinsically involved in the formation of tight junctions and the process of epidermal differentiation normally increases their expression. From our trancriptome analysis we see that they were upregulated by the addition of calcium (KC/K) by 9-fold and 5-fold respectively (Figure S4, Table S3), but both genes were significantly downregulated by VZV infection (Figure 7A). In contrast to Human Papillomavirus, another epitheliotropic virus which downregulates β4 integrin to dysregulate epidermal differentiation [22], VZV infection had no effect on the basal hemidesmosomal proteins (Table S3).

Transcriptome data on the changes in desmosomal genes were verified by qPCR (Figure 7B) and we confirmed that VZV infection reduced the expression of both DSG1 and DSC1 at the protein level by immunoblotting (Figure 7C). In the presence of the VZV gE protein the expression of both DSG1 and DSC1 was reduced in comparison to the mock infected cells. As seen for changes in cytokeratin expression, downregulation of DSG1 and DSC1 was dependent on VZV gene expression, and could be ablated by UV-treatment and inactivation of the VZV viral inoculum (data not shown). Desmosomes provide intercellular adhesive strength required for the integrity of epidermis, and electron microscopy imaging of uninfected (Figure 7D+G), early VZV infection, as denoted by the presence of viral envelopes but not intact virions (Figure 7E+H) and late VZV infection, where virus particles accumulate at cellular boundaries (Figure 7F+I) revealed that desmosomal junctions were no longer observed when the VZV infection was well developed. Addition of phosphonoacetic acid (PAA), which inhibits VZV DNA polymerase and viral replication, also abrogated the down regulation of DSG1 and DSC1 in VZV infected cells as measured by qPCR (Figure 7J–K). Since late but not immediate early viral gene expression is modulated by PAA treatment, the reduction in desmosomal proteins in VZV infected cells may be due to proteins expressed late in the replication cycle.

**Figure 8:**
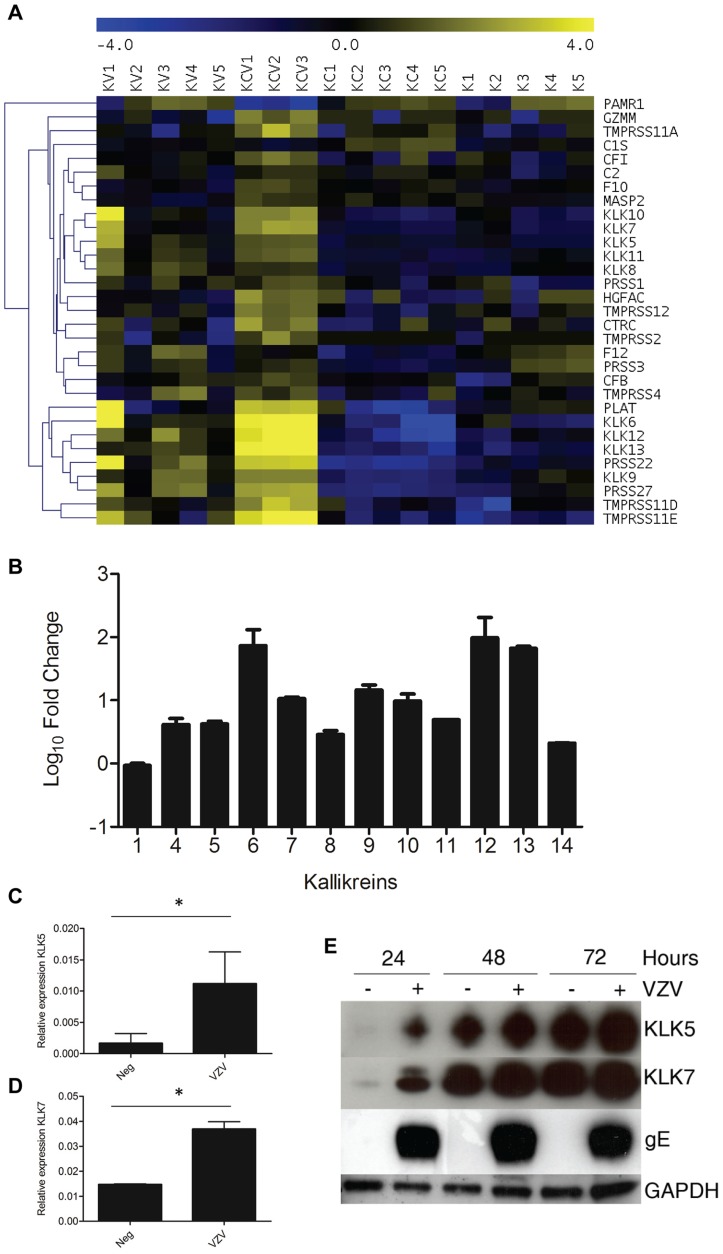
VZV infection increases KLK expression. **A**) Heatmap analysis of transcriptome changes in the serine peptidases and non-peptidase homologues group (IPR001314). A clear upregulation (yellow) of the majority of the genes in this group was observed in all KCV lanes. **B**) The majority of the kallikreins were upregulated in the differentiated keratinocytes compared to the uninfected cells (KCV/KC). Upregulation of KLKs in transcriptome of VZV infected differentiated keratinocytes was confirmed by qPCR for KLK5 and KLK7 (**C–D**). The relative expression of both genes ± standard deviation, normalised to GAPDH is shown for the uninfected and VZV infected samples at day 5 p.i. after the addition of calcium at day3, p values less than 0.05 are indicated (*). Immune blotting for KLK5 and 7 from concentrated supernatants of uninfected and VZV infected keratinocytes at 1–3 days post-differentiation (**E**). GAPDH and gE from the cell lysates was used as a loading control and to show VZV infection respectively.

Serine proteases as a group were significantly enriched in both the VZV infected samples (KC and KV) (Figure 3B–C). A heatmap of the serine peptidases and non-peptidase homologues group (IPR001314) (Figure 8A) displays the differential expression of these genes under our four different conditions. Overall, the majority of the genes are significantly upregulated in the KCV samples, with a similar but less pronounced upregulation also seen in the KV samples compared to K and KC (Table S4). Epidermal serine proteases, such as the tissue kallikreins participate in desquamation, the natural process by which individual corneocytes are shed from the surface of the epithelium [23]. Mutations that reduce the ability of the antagonist to inhibit the activity of the proteases, result in uncontrolled proteolytic activity associated with inflammatory skin conditions e.g. Nethertons syndrome [24]. Our data demonstrates that VZV upregulated the expression of the majority of the kallikrein genes (Figure 8B). The upregulation of KLK5 and −7 was of particular interest due to their role in cleaving DSC1 and DSG1 [25]. The upregulation of these genes by VZV could augment the downregulation of DSG1 and DSC1 thereby further reducing cell-cell adhesion and the strength of the epidermal barrier to withstand mechanical trauma. The upregulation of the KLK5 and −7 genes was validated by qPCR (Figure 8C–D respectively) and the effect of VZV on the KLK5 and −7 proteins was confirmed by immunoblotting in concentrated supernatants from mock and VZV-infected differentiated keratinocytes (Figure 8E).

### The addition of calcium to infected keratinocyte differentiation increases VZV gene expression

**Figure 9:**
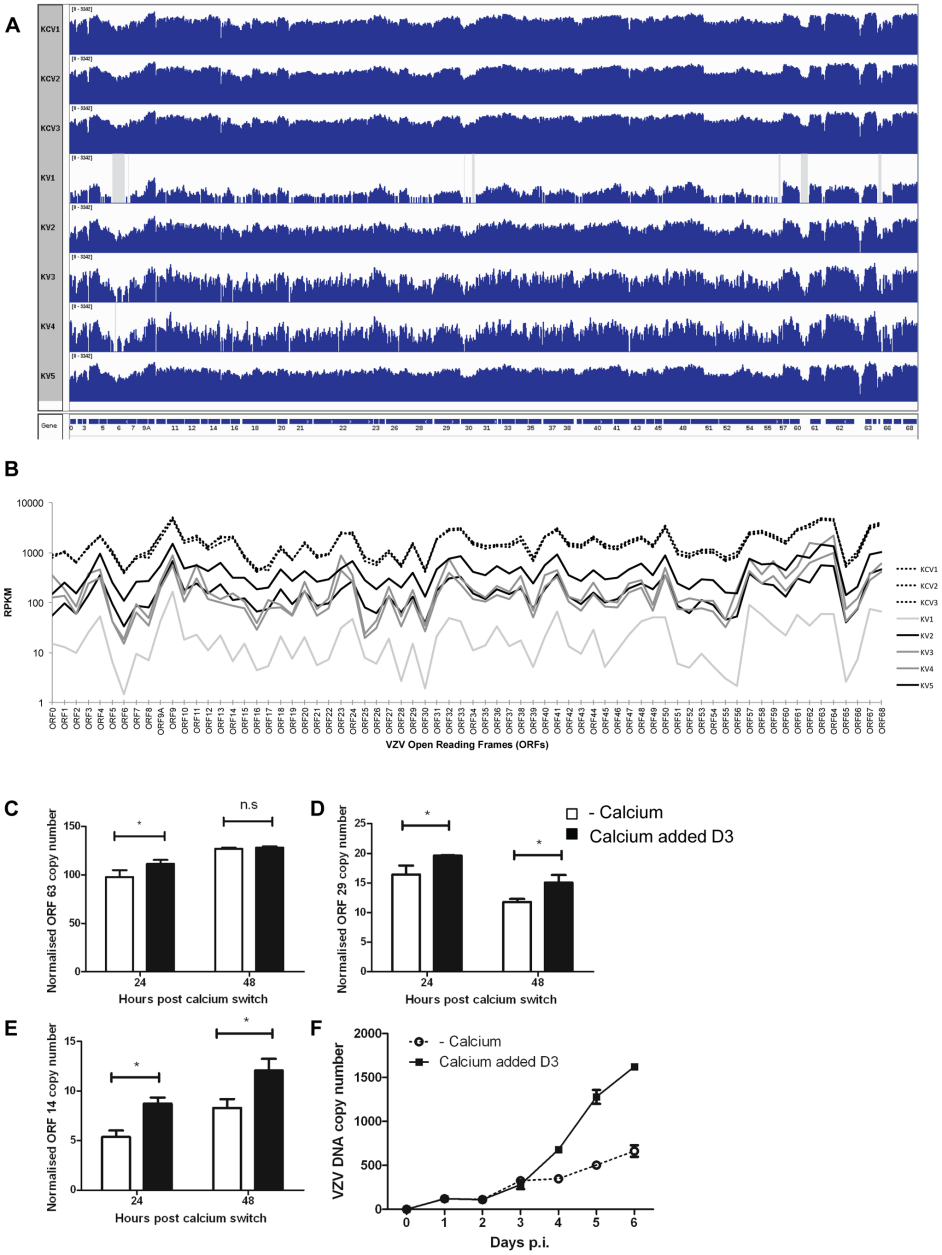
Epidermal differentiation increases VZV transcription. **A**) Coverage plots of viral RNA-seq reads mapped to the annotated VZV pOka genome. The top three panels represent KCV1–3 samples and the lower 5 samples are KV1–5. A schematic representation of the VZV open reading frames is shown below. Each coverage plot uses an identical log scale, illustrating the higher degree of coverage in the KCV samples. Regions with zero coverage are shaded grey. **B**) RPKM values for each of the VZV ORFs across the infected samples. The RPKM values are normalised relative to the sum of human and viral reads per sample. The pattern of expression is similar across all samples with RPKM values for each ORF being greater than 1 in every sample. RPKM values for the KCV samples are less variable than those for the KV samples with a median 9-fold upregulation per ORF (KCV/KV, range 4–15 fold upregulation, all ORFs significantly upregulated with p_FDRs_<0.01). RPKM values for KV1 are noticeably lower than all other samples. At day 5 p.i., 48 hrs after the addition of calcium, the levels of three VZV genes were determined by qPCR and normalised to the housekeeping gene RN5S, no amplification was seen in the mock infected controls (data not shown). The upregulation of KRT10 in the uninfected controls was used as a marker to ensure that the addition of calcium had caused keratinocyte differentiation (data not shown). The expression of (**C**) ORF63 (IE) ORF29 (early) and ORF14 (late) genes was determined (n = 3) and plotted. Expression of ORF29 (**D**) and ORF14 (**E**) was significantly increased after the addition of calcium, *p<0.05 by Student's t-test. **F**) The rate of vDNA replication, as measured by qPCR increases after the addition of calcium (at 48 hrs) in the differentiated cells (n = 3, ± stdev).

RNA-seq enabled analysis of both host and viral transcripts within the same sample. Paired-end reads from VZV infected samples were mapped to the pOKA genome (accession number AB097933). Between 1.4×10^5^–1.2×10^6^ reads were mapped to the VZV genome (Table S1) with the exception of KV1 where the number of mapped reads was at least ten fold lower. Visualisation of the VZV transcripts for all infected samples using IGV (Figure 9A) revealed that all viral genes were expressed in all infected samples, indicating that lytic viral replication was occurring under all conditions. This was also established by electron microscopy examination of the infected cells, which revealed the presence of highly cell associated virus particles in both VZV infected cells treated or untreated with calcium (Figure S6).

Overall, the pattern of VZV gene expression was similar for untreated and calcium treated cells, but the average number of mapped reads was approximately 9-fold higher in differentiated cells (KCV1–3) (Figure 9B). Significantly higher viral expression was observed in the differentiated cells compared to the undifferentiated cells for every viral ORF without exception and regardless of temporal classification (all ORFs up-regulated between 4 and 15 fold; p_FDR_<0.01). Six viral ORFs were significantly changed by the addition of calcium (p<0.01) although only ORF14 (gC) and ORF55 were significant (p_FDR_<0.01) following correction for multiple testing (Table S5) and when KV1 was excluded due to low VZV reads only ORF14 remained significant. The increase in viral gene expression was independently investigated by qPCR analysis for the three temporal classes of herpesvirus genes. No difference was seen in the expression of the IE gene (ORF63) at 48 hrs after the addition of calcium (Figure 9C), however the expression of the early (ORF29) and late (ORF14) viral genes was significantly increased in cells where calcium was added 3 days after VZV infection as per our model (Figure 9D–E). The qPCR data did not reflect the degree of change and increase in VZV gene expression seen in the KCV samples in RNA-seq data in comparison to the KV samples. We were able to demonstrate an effect of calcium on the viral DNA and observed a three-fold increase in VZV DNA as measured by real time PCR after the addition of calcium to infected keratinocytes on day three (Figure 9F). Taken together, our data, whereby calcium induced differentiation of primary keratinocytes increases VZV DNA replication and gene transcription, both of which are required for the production of progeny virions implies that the process of differentiation increases VZV replication, but, we were unable to demonstrate that calcium differentiation induced an increase in the number of VZV particles or replication by either IF, infectious VZV foci or EM (data not shown).

### Pattern of VZV gene expression is altered by keratinocyte differentiation

**Figure 10:**
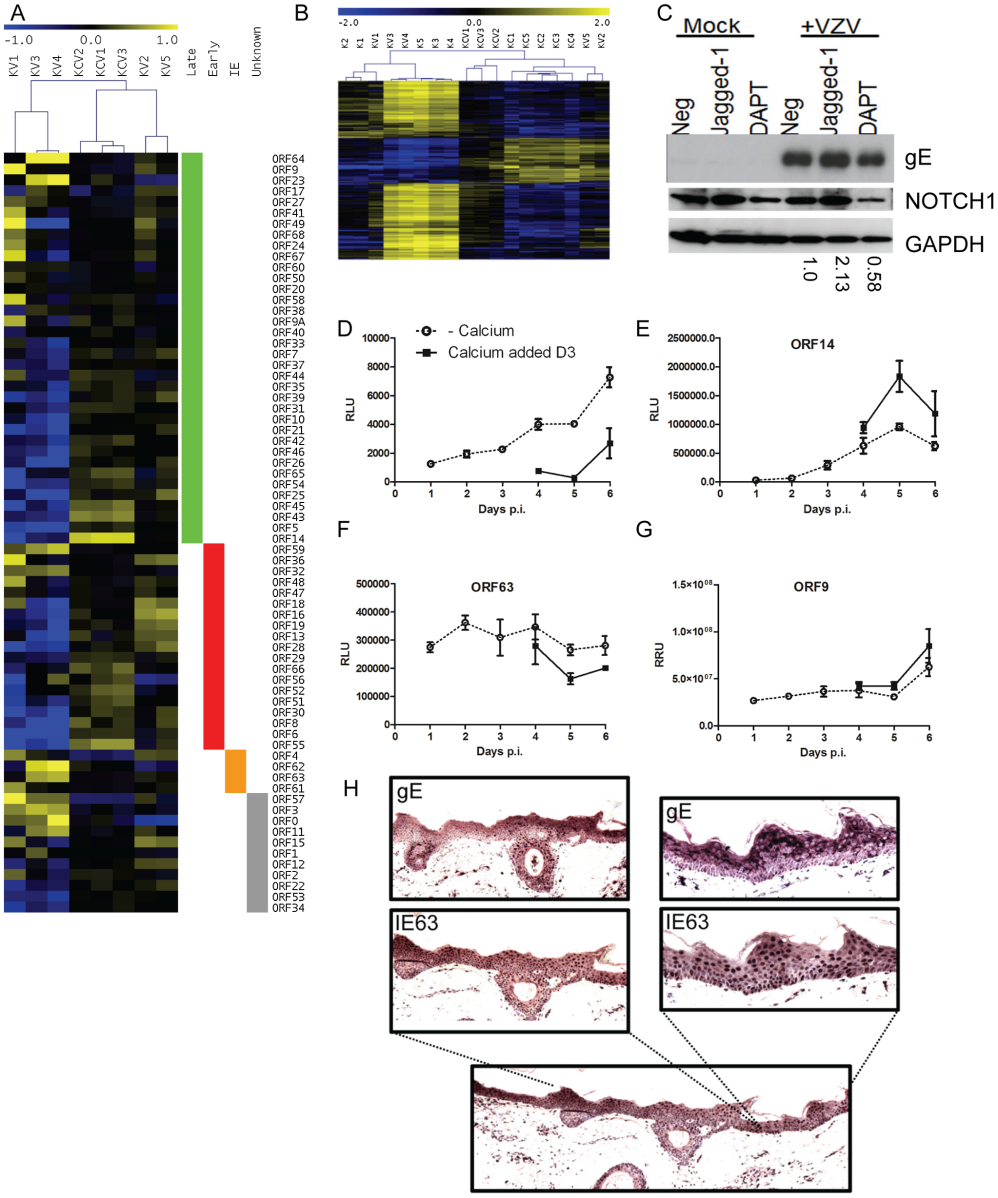
Altered VZV gene expression following keratinocyte differentiation. **A**) Heatmap illustrating VZV ORF expression profiles for the infected samples. RPKM values were calculated for each sample by normalising to viral reads alone and then median-centred for each ORF to highlight relative fold changes across all samples. Samples are hierarchically clustered by Pearson's correlation coefficient. ORFs are ordered by temporal gene expression (i.e. whether they are currently categorised as late, early, immediate-early genes). Log_2_(fold changes) are shown relative to the median RPKM for each ORF. **B**) Heatmap of 1463 human genes altered solely in the KC/K comparison. TMM-normalised CPM values are median-centred for each gene to highlight relative fold changes across all samples. Genes and samples are clustered by Pearson's correlation coefficient. Log_2_ (fold changes) are shown relative to the median CPM for each gene. Samples cluster primarily by experimental condition (i.e. addition of calcium) with the exception of KV2 and KV5 which cluster alongside the calcium-shifted KC samples suggesting these 2 samples had undergone spontaneous contact-induced differentiation. **C**) Primary keratinocytes were infected VZV at an mo.i. of 0.2 and treated with either DAPT [1 µM] or Jagged-1 [50 µM] and harvested for analysis by immunoblotting 48 hrs later. Jagged-1 treatment caused an increase and DAPT treatment showed a decrease in Notch 1 expression respectively as seen in the uninfected keratinocytes. Treatment of VZV infected cells with jagged-1 increased gE expression and DAPT treatment decreased gE expression relative to the untreated VZV infected keratinocytes, the changes in gE were quantified using ImageJ and the numbers below represent the gE expression relative to the untreated VZV infected keratinocytes. Primary keratinocytes were infected with VZV at day 0 with an m.o.i. of 0.2. At day 3 half of the samples were switched to a high calcium media. Duplicate samples were taken every 24 hrs to measure luciferase and/or renilla. Three different recombinant viruses were used **D**) VZV_LUC_ under the control of an IE promoter (ORF4), **E**) ORF14_Luc_ and **F–G**) ORF63_Luc_ORF9_Renilla_. The average relative luciferase/renilla units RLU/RRU for each virus is plotted ± stdev. **H**) Skin explants were injected into the dermis with cell associated VZV (1250 PFU), placed on grids, and harvested at 3 days post infection, fixed and embedded in paraffin. Sections were stained with antibodies against the immediate early viral marker, IE63, and the late marker glycoprotein E (gE). Staining was developed with the chromogen VIP. No staining was seen in the uninfected controls (data not shown). Although epidermal infection was evident, this model did not demonstrate epidermal blistering. VZV staining is present throughout the epidermis and the expression gE was more apparent in the higher layers of the epidermis.

In common with other members of the herpesvirus family, VZV gene expression occurs in a temporally regulated cascade, which can be categorized as; immediate early (IE), early (E) and late (L). When viral reads were normalised separately to human reads, distinct patterns of relative viral gene expression were observed for the undifferentiated (KV) and differentiated (KCV) samples within each sample (Figure 10A). A high degree of agreement was observed between the differentiated samples (KCV1–3) indicating that late viral genes were more highly expressed than immediate early genes in the differentiated samples. Undifferentiated samples KV3 and KV4 also showed good agreement, where in contrast to KCV1–3, the immediate early genes were more highly expressed than the late genes. However, samples KV2 and KV5, despite not having been treated with calcium, had similar viral gene expression patterns to the differentiated cells (KCV1–3). Analysis of 1463 host genes which are differentially expressed after treatment with calcium but not changed by VZV infection (i.e. representative of keratinocyte differentiation) showed clustering of KV2 and KV5 host gene expression profiles with the calcium-shifted differentiated keratinocyte samples KCV1–3 (Figure 10B). This finding was supported by Spearman's rank correlation coefficient (Figure S3) and principal component analysis of host gene expression profiles (Figure S3), which again clustered samples KV2 and KV5 with the calcium treated samples. Since KV2 and KV5 were not treated with calcium, it is likely that these replicates underwent spontaneous differentiation, something that is known to happen when primary keratinocytes contact each other [26]. These data illustrate the impact of keratinocyte differentiation on VZV gene expression as distinct from the effect of calcium. To test our hypothesis that it is differentiation and not the addition of calcium that is responsible for the increase seen in VZV gene expression, the NOTCH pathway was activated by the addition of the agonist, jagged-1 and inhibited by the addition of DAPT. The canonical NOTCH pathway acts as a switch between the basal and suprabasal genes and is a key regulator of keratinocyte differentiation and its activation causes stem cells to exit their niche and start the process of terminal differentiation [27]. NOTCH activation and ablation influenced gE expression as measured by western blotting (Figure 10C). gE expression was increased in comparison to the untreated keratinocytes, when the NOTCH pathway was activated. Conversely a decrease gE protein expression was observed relative to the untreated keratinocytes when the NOTCH pathway was ablated.

To examine the effect of cellular differentiation on the regulation of VZV genes, we used a recombinant viruses expressing either luciferase or renilla reporter cassettes under various VZV promoters (Figure 10D–G). Keratinocytes were infected with one of the three viruses as per our model and the reporter activity measured over the course of 6 days. Reporter activity for ORF4, ORF14 and ORF9 increased over the timepoints taken whereas ORF63 remained relatively level. In all viruses, the reporter activity was altered by the addition of calcium at day 3 post infection. With the reporter driven by an immediate early promoter (Figure 10D and F) there was a decrease in its activity after the addition of calcium whereas the converse was true for the reporters driven by the late promoters (Figure 10E and G). The increase in ORF4 promoter activity in undifferentiated cells and not in the calcium treated cells as well as the increase in the late viral promoter activity and viral early and late gene products in the calcium differentiated cells suggests that there was a relative block to viral replication which was overcome by keratinocyte differentiation.

To investigate if the expression of the VZV proteins is affected by differentiation in the epidermis, skin organ cultures were intradermally injected with VZV to model skin infection and stained for IE and late proteins 3 days post infection (Figure 10H). Although epidermal infection throughout the section was evident, the explant model did not demonstrate epidermal blistering unlike the *in vivo* archival specimens (Figure 6B–G). VZV IE63 staining was found predominantly in the nuclei throughout the epidermis and the expression of late viral glycoprotein gE was largely cytoplasmic with increased expression of gE seen in the uppermost layers of the epidermis. Together with the observed differential gene expression as keratinocytes differentiate under calcium, these results indicate that VZV gene expression is tied to regulated keratinocyte differentiation, with a switch from more efficient early gene expression at the undifferentiated stage to late gene expression as differentiation ensues.

## Discussion

Keratinocytes, the predominant cell type found in the epidermis, are a major target of VZV replication in skin. The calcium switch method delivered a dynamic model of epidermal differentiation which can be manipulated to allow investigation of viral and host interactions during synchronized keratinocyte differentiation without the presence of other cell types.

A number of alternative systems have been described to investigate the biology of skin, including 3D raft cultures and explants [20], [28]. A further model, using SCID-hu mice, has previously been successfully used to study the infection dynamics of VZV *in vivo* [13]. Both the 3D raft cultures and the explants show a greater degree of differentiation, formation and definition of the structural layers characteristic of skin in contrast to the calcium shift model utilised in this study. Furthermore, the explant system also contains the presence of specialised structures, such as the sebaceous glands and hair follicles that are absent from raft cultures and the monolayer system. However neither the explant nor the SCID-hu mouse model was suitable for this study as neither system can be manipulated experimentally to allow the effects of differentiation on VZV gene expression to be examined. In contrast, the development of 3D organotypic rafts whilst offering a valid alternative model to allow investigation into the effects of virus infection on cellular differentiation and vice versa was not found to be reliable enough, particularly in the presence of virus, for use in the RNA-seq part of our study.

The model has certain disadvantages e.g. the genes associated with the stratum lucidum and stratum corneum are not expressed. The titre of virus produced in primary keratinocytes is at least 2 logs lower than is seen in MeWo cells and remains highly cell associated as we saw in our EM images. Thus although we were able to show that calcium induced increases in gene expression and viral genome replication, these were not associated with an increase in the formation of infectious particles. Nonetheless, our data clearly demonstrate that, in common with the archetypal alphaherpesvirus HSV-1 [29] and previous observations [10], [20], VZV appears to preferentially infect undifferentiated keratinocytes. In epithelia, undifferentiated keratinocytes are localised to the stem-cell rich basal layer of the epidermis and adnexal structures. Our findings are consistent with histological evidence of early Herpes Zoster lesions that shows VZV infecting the stem-cell rich isthmus region of the hair follicle [30], following reactivation from latency and prior to the onset of the epidermal infection or distinctive cutaneous rash. By adding calcium to VZV infected undifferentiated keratinocytes our model therefore mimics the likely sequence of events in the skin, with infection of less differentiated basal cells followed by differentiation of infected cells. The fact that VZV spreads more easily in undifferentiated cells fits with the model of cell associated virus inhabiting basal epidermal layers while cell free virus in the upper epidermis is necessary for transmitted infection [31]. Virus adapted for cell to cell spread in the basal epithelium has been shown to differ from cell-free virus. For example, VZV can spread efficiently cell to cell in MeWo monolayers despite low levels of envelope glycoprotein C [32] a protein that is necessary for the formation of cell free virus and VZV replication in skin [13], [33]. This fits with our results showing increased late viral protein expression in the suprabasal layers where assembly of cell free virus occurs [31]. One model to explain our results may therefore be that the generalised increase in viral gene and protein expression seen with keratinocyte differentiation is directed to maturation of cell-free virions from the immature viral particles formed in the basal layers, rather than a large increase in numbers of virions. Over time, as keratinocyte maturation continues, high titers of cell-free virions accumulate in the growing blister.

The blister lesions formed in VZV infections are by and large discrete, although coalescence of mature lesions may occur. While production of interferon by bystander cells has been shown to limit viral spread in the skin [34] it is also possible that cell-to-cell spread is limited by the physical barriers associated with keratinocyte differentiation, thus explaining the reduced spread of virus observed in differentiated cells. Alternatively, differentiation may result in the loss of a cellular receptor rendering keratinocytes less permissive to VZV infection. Of the three known putative cellular receptors for VZV, mannose 6 phosphate receptor, reported to be critical for the accumulation of cell free virus in vesicles and Heparan sulphate are expressed only in basal layers and the expression of both are lost as keratinocytes differentiate [31] [35]. Both are therefore candidates for viral spread in less differentiated but not differentiated cells. In contrast, expression of the third putative receptor, insulin degrading enzyme (IDE), increases as keratinocytes differentiate (Jones M. unpublished data).

Notwithstanding the reduced cell-to-cell spread in differentiated keratinocytes, our analysis revealed a quantitative increase in viral gene and viral DNA expression after calcium induced differentiation and by manipulation of the NOTCH pathway as well as by calcium and contact induced differentiation we were able to show that this was dependent on differentiation and not just the addition of calcium to our cells. As previously outlined, these results are not necessarily contradictory but are consistent with a model by which the virus is able to enter and spread in undifferentiated keratinocytes, but once infected, optimal replication and production of mature virions requires the presence or loss of cellular factors present as cells differentiate. Although VZV does not persist in the skin, it shows a pattern common to other skin tropic viruses. Human Papillomavirus (HPV) which maintains its genome as a stable episome in basal cells until differentiation of the host cell occurs [36] and the gamma-herpesviruses Kaposi Sarcoma Herpesvirus (KSHV), whose lytic cycle is also known to be activated by keratinocyte differentiation [37].

In our model, expression of all VZV ORFs was evident in both untreated and calcium treated keratinocyte samples, confirming that VZV undergoes full lytic infection in both conditions. However, the distinct differences in the pattern of expression were clearly apparent, with relatively more immediate early genes (ORFs 4, 62 and 63) in the undifferentiated cells and relatively more late genes (e.g. viral glycoproteins) expressed in the keratinocytes that had undergone synchronized differentiation. The apparent association of each condition with IE or late viral gene expression suggests that the state of the host cell may impact on the regulation of the molecular switch controlling the classical temporal pattern of herpesvirus gene expression. Though not definitive proof, this hypothesis is supported by our observations. First, the expression of the VZV promoters in the recombinant viruses clearly establishes differential activity of the reporters in response to calcium induced keratinocyte differentiation. This block in production of early and late viral proteins in undifferentiated keratinocytes is consistent with a requirement for cellular factors present in differentiated cells to regulate the switch to early/late gene expression. Secondly, the expression of gC, which is essential for viral replication in skin [13], was significantly greater in the calcium switched cells. This result corroborates previous work where increased gC expression was observed in MeWo cells treated with hexamethyl bisacetamide (HMBA), a known inducer of cellular differentiation [32]. Finally, using immunohistochemistry we were also able to show that another late protein, gE, was more highly expressed in the upper layers of the epidermis in explants.

Of interest, several VZV ORFs which, based on their HSV-1 orthologues are presumed to be late genes, were more highly expressed in the undifferentiated cells. These include ORFs 17 (virion host shutoff), 64 (tegument U_S_10), 46 (tegument U_L_14), 27 (nuclear phosphoprotein U_L_31), 60 (gL) and 23 (capsid). Further work is required to determine whether these findings reflect true differences in VZV temporal gene expression or whether they are indicative of keratinocyte specific differences in the gene expression patterns.

Analysis of the host transcriptome confirmed VZV does not drive keratinocyte differentiation. Instead the virus clearly alters the normal pattern of gene expression associated with differentiation, generating a signature associated with skin blistering, which itself is a characteristic feature of VZV disease. VZV downregulated or prevented the expression of the suprabasal genes, KRT1 and KRT10, but was not shown to alter the expression of other differentiation markers such as involucrin. *KRT1 and* KRT10 are known to play a role in maintaining the integrity of the epidermis and are mutated in other blistering diseases such as epidermolytic ichthyosis [38]. KRT10 has also been shown to inhibit proliferation and cell cycle progression of basal keratinocytes [39], [40] and its loss is also associated with increased cell turnover [41]. However, through its interactions with desmosomes, KRT1/10 form a dynamic scaffold in the cell and play an important role in maintaining epithelial structure [42]. Autoantibodies to desmosomal proteins, DSG1 and DSC1 proteins are a hallmark of blistering skin conditions including pemphigus foliaceus and IgA pemphigus [43]. As a group, the desmosomal genes, especially DSG1 and DSC1 were identified as being significantly altered by VZV infection while other junctional proteins were unaltered. These findings support the notion that the interaction of VZV with keratinocytes drives the pathognomonic blistering phenotype. The accompanying upregulation of the serine proteases by VZV may contribute to the observed reduction of DSG1 protein as well as inducing a desquamative phenotype, which may promote the dissemination of the virus.

An unusual finding which is not typical of blistering disorders was the upregulation of the mucosal cytokeratins 4 and 13, a phenotype previously observed in keratinocytes with impaired SMAD 2, 4 and 7 signalling [44]. Jones *et al.* previously observed such changes in the TGF beta pathway in a VZV infected SCID-hu mouse model [45]. Although we observe the same gene pattern changes as Buschke *et al.* in that we observed downregulation of KRT1/10/DSG1 and upregulation of KRT4/13, we did not find a significant enrichment of the TGF-beta pathway as a whole. It is possible that our transcriptome analysis, which was a single snapshot late on in VZV infection, failed to detect early signalling changes responsible for KRT4/13 changes. Alternatively, VZV may act via different pathways in this system.

Other observed changes in cytokeratin expression which do not usually form part of a blistering signature include the upregulation of KRT15 and KRT19 which are associated with stem cells in adnexal skin compartments, a region which histologically is positive for VZV early on in infection. KRT15 is also associated with wound healing, but other markers of keratinocyte activation in wound healing (e.g. KRT6, 16 and 17) [46] were unaltered in our transcriptome data.

In summary, we have shown by combined analysis of host and pathogen gene expression at a single time point that VZV gene expression is linked to keratinocyte differentiation. VZV replication, in turn, alters the structure of stratified squamous epithelium, driving a blistering, desquamative phenotype to form the typical skin vesicles, which are essential to VZV pathogenesis. The major functional groups studied in the manuscript i.e. the alterations affecting cytokeratins, desmosomes and proteases, are controlled by a number of regulatory pathways and further work is underway to untangle the complex molecular interactions between VZV and keratinocyte differentiation. While the data presented here are only a snapshot of this complex process, they provide a roadmap for further exploration of how VZV interacts with a target cell central to its pathogenesis.

## Materials and Methods

### Ethics statement

Human skin from cosmetic reductive surgery was obtained with written informed consent under the approval of the East Central London Research Ethics Committee 1 (10/H0121/39).

### Cells

Neonatal primary human epidermal keratinocytes (HEKn, Life technologies, Paisley, UK) were cultured on mouse collagen IV (0.67 µg/cm^2^ BD Biosciences, Oxford, UK) coated surfaces in keratinocyte defined media containing epithelial growth factor (KDM and EpiLife, Life technologies). Differentiation was induced by shifting the cells to a high calcium media containing [1.2 mM] calcium chloride. Neonatal Human Dermal Fibroblasts (HDFn, Life technologies) were cultured in medium 106 supplemented with low serum growth supplement (Life technologies). MeWo cells were cultured in MEM (Sigma, Dorset, UK) supplemented with 10% (w/v) FBS and 1% non-essential amino acids. nTERTs cells were cultured in 3∶1 DMEM∶Ham's F12 supplemented with 10% FBS, 1% L-glutamine (200 mM) and Ready Mix Plus (0.4 µg/ml hydrocortisone, 5 µg/ml insulin, 10 ng/ml EGF, 5 µg/ml transferrin, 8.4 ng/ml cholera toxin and 13 ng/ml liothyronine). All uninfected cells were cultured at 37°C, 5% CO_2_.

### Skin explant culture

Segments of human skin (less than 2 cm^2^) were intradermally inoculated with approx. 1×10^5^ infectious units of cell-free virus and cultured at the air-liquid interface in Dulbecco's modified Eagle's and Ham's F12 medium (3∶1), supplemented with 10% foetal bovine serum, 1% L-glutamine, and supplemented with RM+ (0.4 µg/ml Hydrocortisone, 5 µg/ml Insulin, 0.01 µg/ml EGF, 0.0084 µg/ml Cholera toxin, 5 µg/ml Transferrin and 0.0013 µg/ml Lyothyronine) for 10 days. Mock-injected segments were cultured in parallel as a control. Skin samples were fixed in 4% paraformaldehyde and embedded in paraffin.

### Viruses

Infections for transcriptome experiments were carried out using pOka and validation experiments were carried out using a strain named THA, a low passage clade 3 clinical isolate. The VZV ORF23 GFP, expressing an N terminal tag to the capsid OR23 protein, has been detailed previously [47]. A recombinant VZV expressing luciferase (VZV_LUC_) driven by ORF4 promoter was developed by cloning the ORF4 promoter upstream of the luciferase gene in the vector PGL3 (Promega Corp), followed by PCR amplification of the entire cassette and cloning into the pOka based cosmid pspe23 at the unique AvrII site located between OR65 and 66. This was then developed into recombinant VZV with additional pOka cosmids as described previously [48]. The ORF14-luciferase virus reports luciferase as a T2A directed ribosome skipping motif fusion protein. Luciferase was amplified by PCR from the plasmid pGL3basic (Promega Corp) using the primers 5′ GAGGGATCCGGTTCCGGAGAGGGCAGAGGA AGTCTGCTAACATGCGGTGACGTCGAGGAGAATCCTGGCCCAATGGAAGACGCC AAAAACATA-3′ and 5′ AATTCGAATTCGCGCGCAGATCTTTACACGGCGATCTTTCCGCCCTTCTTGGC-3′. The resulting fragment was digested with EcoRI and BamHI and cloned into the vector pmCherryC1 cut with EcoRI and BglII (underlined in primers), resulting in plasmid pmCherryT2Aluc which contained mCherry fused in frame to luciferase, separated by the T2A 22 amino acid ribosome skipping motif. The expression of functional luciferase (containing a single residue added to the amnio terminal end) and mCherry (with a 21 amino acid T2A C terminal addition) were confirmed in plasmid transfected HEK293T cells (data not shown). The plasmid was cut with EcoRI and BglII (both sites downstream from the T2A luciferase gene) and a zeomycin resistance cassette was inserted following its generation by PCR with primers to add bglII and EcoRI flanking sequences (underlined in the following primers 5′ AGATCTAGATCTCGAGTAATGGAACGGACCG TGTTGA C-3′ and 5′ – GCTGAC GTCGACGAATTCTGATCACTCAAGTTTCGAGGTCGAGGTG 3′). The resulting plasmid was used as the template for the PCR amplification of the entire T2A-Luciferase-Zeo cassette using primers with 40 bp flanking homology arms to allow recombination into ORF14 in the pOka BAC, so it was an in frame fusion with the terminal residue of gC (ORF14): using the primers gClucF2 5′ CTTATCGCAGTTATC GCAACCCTATGCATCCGTTGCTGTTCAATGGACGAGCTGTACAAG-3′ and gClucR2 5′ ATAAAATGATATACACAGACGCGTTTGGTTGGTTTCTGTCTCGAGTATGATCAG TTATC 3′. The PCR product was amplified, gel purified and transformed for recombineering into pOka BAC [49] using pGS1783 bacterial host (a kind gift of Gregory Smith Northwestern University IL), a VZV pOka BAC detailed previously [50] and recombineering methods detailed by [51]. Chloramphenicol resistant BACs also showing zeomycin resistance were validated for DNA integrity and correct insertion into ORF14. Virus was derived by cotransfection of the BAC purified DNA into MeWo cells as previously described [50]. VZV containing both ORF63-T2A luciferase and ORF9-T2A renilla reporters were generated similarly. Renilla gene was first PCR amplified to add the T2A motif to the N terminal end using the following primers and the template pSV40 RL (Promega Corp) 5- AGAGGATCC GGTTCCGGAGAGGGCAGAGGAAGTCTGCTAACATGCGGTGACGTCGAGGAGAATCCTGGCCCA***ATG*** *ACTTCGAAAGTTTA TG -3′* *and* *5′* GAATTCGAATTCTGTTCATTTTTGAGAACTCGCTCAA-3′. The EcoRI and BamHI digested product was cloned into pEGFP-C1 cut with bglII and EcoRI, to generate the plasmid pEGFPT2ARen. A kanamycin resistance cassette was amplified from pEPS kan 2 [51] using primers that added flanking repetitive sequences to allow subsequent recombineering removal of the cassette, using 5′ AGATCTAGATCT *AGGATGACGACGATAAG* ***TAGG G-3′*** and ATTCGAATTCCGATGAACTCAGTAGCATTATTGTTCATTTTTGAGAACTCGCTCAACGAACGATTTGATATCAACCAATTAACCAATTCTGATTAG-3′ primers. The BglII and EcoRI digested cassette was cloned into the unique EcoRI and bglII sites downstream of the renilla cassette in pEGFPT2ARen. This was used as a template for PCR amplification of the entire T2A-renilla kanamycin cassette with primers that added 40 bp homology arms to ORF9, so as to place the cassette as a fusion to the C terminal residue of ORF9 (5′- AGTAGGGCCCGTTCGGCATCAAGAACTGATGCGCGAAAAATG GAC GAGCTGTACAAG -3′ and 5′- TTATACATAATACCGGGTAAACCGTTACTGCGTAATTAACTCGAGTATGATCAGTTATC 3′

Recombinants of pOka BAC containing the cassette were selected based on gain of kanamycin resistance. A second recombination event was induced concordance with ISce induction [51] to remove the kanamycin cassette, and transformants were screened for loss of kanamycin. The resulting pOka BAC containing the ORF9-T2A renilla fusion was subjected to a third induced recombination following transformation with a T2A luciferase-Zeo cassette, amplified using primers to add flanking 40 bp homology arms to enable recombination to the C terminus of ORF63 (5′ GGAAAATATCAACATAAAATATATCATCGTAAAAATTCGAGTATGATCAGTTATC 3′ and 5′ GCTCCCGTCATAGCAAATACAAAGACAATTATTAGCGTAATAATGGACGAGCTGTACAAG 3′. Zeomycin resistant positive transformants were screened for integrity and correct insertion using sequencing. Finally, a kanamycin resistant cassette was inserted into ORF71 to prevent rescue of the ORF63-T2A luciferase cassette by the duplicated gene using a fourth induced recombination. Virus was derived from the BAC on MeWo cells and replicated efficiently as wild type (data not shown). The virus derived from the BAC contained deletion of ORF71, a fusion of ORF63 to T2A luciferase and a fusion of ORF9 to Renilla.

### VZV growth and infections

All viruses were cultured and used at less than 20 passages. Infected cell preparations were first treated with mitomycin C (0.05 mg/ml for 3 hrs) prior to freezing and subsequently titered on Mewo cells; we routinely achieved viral titres of greater than 1×10^6^ pfu/ml. Cell-free VZV was generated by a rapid freeze (liquid N_2_) and thaw (37°C), followed by removal of cellular debris by low speed centrifugation. In comparison to the cell-associated VZV, titres for cell-free virus were reduced by 1–3 logs and we achieved titres of approximately 1×10^4^ pfu/ml. For UV inactivated VZV, cell-free VZV supernatants were treated for 20 min at 150,000 J/Cm^2^ using a Stratalinker UV Crosslinker (Stratagene). For the transcriptome and subsequent confirmatory experiments, 3×10^4^ HEKn cells per well of a 6 well dish were plated out at day 0 and left for 48 hrs, one well was then used to calculate the cell density and other wells were then inoculated with cell-free virus at an m.o.i of 0.2 as calculated by viral titration on Mewo cells. The cells were cultured at 34°C, 5% CO_2_ for 3 days before either maintaining the changing the calcium at [0.6 µM] or increasing to [1.2 µM]. Parallel wells were used to confirm the percentage of cells infected as measured by flow cytometry. For immunofluorescence, the experiments were carried out on a coverslip in a 24 well plate using 5×10^3^ cells, which were infected with an m.o.i. of 0.2. as above. other details are as above. Infection of nTERTs for PCRs and westerns were carried out in a 6 well plate, with 0.25×10^6^ cells left for 24 hrs before infection with an m.o.i. of 0.2. For immunofluorescence, 1×10^4^ nTERTs were plated on a coverslip and infections carried out at an m.o.i. of 0.2 for 72 hrs before fixation with 4% PFA.

HEKn were seeded onto a de-epidermilised dermis (DED) and cultured at the air-liquid interface, as previously described [52]. Rafts were intradermally inoculated with approximately 1×10^5^ infectious units of cell-free virus nine days post-lifting. Mock-injected segments were cultured in parallel as a control. Five days post-infection, all cultures were fixed in 4% PFA and embedded in paraffin.

### Library preparation

Total RNA was extracted using TRIzol reagent (Life technologies) and cDNA libraries were prepared using reagents and protocols supplied with the mRNA seq kit (Illumina, Essex, UK). Briefly, poly-A tailed RNA was purified from 10 µg of total RNA using oligodT beads. The purified RNA was fragmented chemically and cDNA was synthesised using Superscript II (25°C for 10 minutes, 42°C for 50 minutes, 70°C for 15 minutes; Life technologies) primed with random primers supplied in the kit (Illumina). Unique adaptors were ligated to the cDNA and 200 bp fragments were size selected by agarose gel purification. Libraries were validated by using a DNA high sensitivity chip (Agilent, Cheshire, UK) and quantified by Qubit analysis using the Quant-iT dsDNA HS Assay (Life technologies).

### Illumina sequencing

Libraries were sequenced with a 36 bp paired end read using a GAIIx sequencer (Illumina). Each library was loaded onto a sequencing chip at a concentration of 16pM. The library was amplified and the Read 1 sequencing primer was hybridised using Paired end Cluster station reagents version 1 and 2 (Illumina). The paired end module (PEMx) was attached to the GAIIx sequencer for Read 2 preparation. Each run was quality controlled by assessment of the Phix sequencing control, loaded at a concentration of 6pM per chip.

### Computational analysis

Solexa sequencing and pipeline analysis was performed by J.Porwisz (UCL Genomics), generating FASTQ files for each sample using GenomeAnalyzer pipeline v1.4 (Illumina) and CASAVA v1.0 (Illumina). Paired end reads were mapped to host and viral genomes (*Homo sapiens* (release 37) reference sequence (GRCh37/hg19); pOka sequence (pOka, GenBank reference AB097933)) using BowTie [53], TopHat [54], SAMtools [55]. Duplicate reads were then removed using PicardTools (http://picard.sourceforge.net) and read counts per gene generated using HTSeq-count (http://www-huber.embl.de/users/anders/HTSeq). R/BioConductor [56] were used to import the mapped count data and the edgeR package used to normalise the data and generate lists of differentially expressed genes. Specifically, a filtering step was applied remove low expression genes with fewer than 1 count per million (CPM) in at least 3 samples. Counts were then normalised using a trimmed mean of M-values [57] and fitted to a negative binomial generalised log-linear model (GLM), using empirical Bayes tagwise dispersions to estimate the dispersion parameter for each gene [58]. Differentially expressed genes were identified using GLM likelihood ratio tests applying a FDR significance cut-off of 0.01, unless otherwise stated. The gplots R library was used to construct heatmaps (http://cran.r-project.org/web/packages/gplots/index.html). Functional classification of genes was performed using the DAVID online database (http://david.abcc.ncifcrf.gov/home.jsp) [16]. Gene expression heatmaps were generated using the MEV software suite [59], [60] (http://www.tm4.org/mev.html). Integrative Genomic Viewer (IGV) was used to produce viral genome coverage plots [61]. The Venn diagram in figure 2B was generated using the online tool VENNY (http://bioinfogp.cnb.csic.es/tools/venny/index.html).

### Real-time semi-quantitative reverse-transcription-PCR

3 µg of total RNA was DNaseI treated before being reverse transcribed using MMLV reverse transciptase and random hexamer primers (Promega, Southhampton, UK). qPCR was performed using a Rotorgene 3000 cycler (Qiagen, Manchester, UK) using 500 ng cDNA, specific primers [0.4 µM] (Text S1) and SYBR green master mix (Qiagen). Each gene was normalised to a housekeeping gene (GAPDH or RN5S) and relative expression shown as 2^−ΔΔCt^ [62].

### Flow cytometry

HEKn were fixed in 4% PFA, blocked in 10% Goat serum, washed in permeabilisation buffer (eBiosciences, Hatfield, UK) and incubated with the anti-VZV-FITC (Millipore, Watford, UK) for 30 min in the dark. Cells were subsequently washed in permeabilisation buffer, resuspended in PBS, processed by FACS Calibur and analysed using FloJo (v7.6.5).

### Western blotting

Cell pellets were lysed in whole cell lysis buffer (20 mM HEPES KOH (pH 7.4), 50 mM NaCl, 2%w/v NP40, 0.5%w/v NaDeoxycholate, 0.2%w/v SDS, 1 mM NaOrthovanadate, 1 mM EGTA pH 7, 10 mM NaF, 1 mM PMSF, protease inhibitor cocktail (Sigma-Aldrich). Protein concentration was determined by BCA Assay (Thermo-Fisher Scientific, Loughborough, UK). Samples were added to 4× Gel loading buffer (Life Technologies), DTT (0.083M), heated to 70°C for 5 min. Secreted proteins were concentrated from supernatents using Amicon Ultra 10K filters (Millipore) and equal volume of concentrated supernatents were added to 2XSDS loading buffer, heated to 95°C for 5 min. Samples were resolved on a 4–12% Bis-Tris gel (Life technologies, Paisley, UK) and transferred to a nitrocellulose membrane and blocked in 5% PVP, 0.5% FBS. Membranes were incubated with antibodies (Text S2) and detected by ECL plus (GE, Buckinghamshire, UK).

### Immunofluorescence

HEKn grown on coverslips were fixed in 4% PFA for 20 min at RT before being washed in PBS, incubated in NH_4_Cl [10 mM] for 10 min before being permeabilised with 0.05% (w/v) Triton X-100 on ice for 5 min. Cells were blocked with 3% BSA and incubated with the primary antibody (1∶100) (Text S2) for 1 hr, followed by the Alexa Fluor secondary antibody (Life Technologies) (1∶1000) for 1 hr. Cells were mounted in Prolong gold (Life Technologies) and visualized on a Zeiss Axiovision. Images were analysed using AxioVision Rel. 4.8 and ImageJ. Statistical analyses were performed using Prism (GraphPad Software).

### Immunohistochemistry

5 µM paraffin-embedded sections were processed using conventional techniques. Antigen retrieval was performed by heat-treatment of deparaffinised sections in 10 mM citrate buffer pH 6.0. Sections were treated with 3% hydrogen peroxidase and biotin blocked (Vector Laboratories, Peterborough, UK) prior to onset of immunostaining. VZV antigen was amplified using Universal Elite ABC kit in conjunction with M.O.M Biotinylated Anti-Mouse IgG (Vector Laboratories) and visualised using fluorophore tyramide amplification reagent (Perkin Elmer Life Sciences, Buckinghamshire, UK). Nuclear staining was visualised by staining with Hoechst 33342 (Life technologies). Sections were mounted in Immunomount (Thermo-Fisher Scientific, Loughborough, UK) and images were captured using a Leica epifluorescence microscope.

### Electron microscopy

Cells were fixed in 0.5% glutaraldehyde in 200 mM sodium cacodylate buffer for 30 min, washed in buffer and secondarily fixed in reduced 1% osmium tetroxide, 1.5% potassium ferricyanide for 60 min, washed in water and stained overnight in 0.5% Mg Uranyl acetate. The samples were then embedded flat in the dish in Epon resin. Ultrathin sections (typically 50–70 nm) were cut parallel to the dish stained with Reynold's lead citrate and examined in a FEI Tecnai electron microscope with CCD camera image acquisition.

### Plaque assays

Plates were fixed at time points post infection with 4% paraformaldehyde and stained by immunohistochemistry using a mixed VZV mAb (Meridian Life Sciences, Memphis, US), followed by and biotin (Vector labs, Peterborough, UK) streptavidin (Jackson ImmunoResearch, PA, US) amplification. Plaques were visualised using Fast Red TR salt (Sigma, Dorset, UK) and images of stained plaques were digitally captured and counted using the ViruSpot Reader (AID GmbH).

### Luciferase assay

Keratinocytes infected with VZV_Luc_ were lysed and processed for luciferase activity using the luciferase or dual-luciferase reporter assay system (Promega) according to the manufacturer's protocol.

### Viral DNA extractions

vDNA was extracted using the Qiagen DNeasy blood and tissue kit and vDNA copy number was determined by real time PCR for the VZV ORF29 gene, normalized to KRAS (Text S1) using a standard curve.

### Accession numbers for human and VZV genes

#### Cellular gene name (UniProtKB/Swiss-Prot)

KRT1 (P04264), KRT10 (P13645), DSG1 (Q02413), DSC1 (Q08554),

KRT15 (P19012), KRT5 (P13647), KRT14 (P02533), KRT4 (Q6PIN2), KRT13 (P13646)

KRT19 (P08727), KLK5 (Q9Y337), KLK7 (P49862), CD29 (P05556), CD34 (P28906)

CD200 (P41217), IVL (P07476)

#### Virus gene name (UniProtKB/Swiss-Prot)

ORF14 (Q4JR11), ORF63 (Q77NN7), ORF4 (Q4JQX1), ORF68 (Q9J3M8), ORF17 (Q4JQV8), ORF64 (Q4JR15), ORF46 (Q4JQS9), ORF27 (Q4JQU8), ORF60 (Q9J3N1), ORF23 (Q4JQV2)

## Supporting Information

Figure S1 **Increasing calcium concentration induces keratinocyte differentiation.** Primary human keratinocytes grown either in low calcium [0.6 mM] or high calcium [1.2 mM] cultures were processed for total RNA and protein extraction. The increase of CaCl_2_ concentration to 1.2 mM initiated cellular differentiation of human keratinocytes beginning at 24 hrs, as detected by qPCR for (**A**) *KRT15*, a basal layer gene which was downregulated by the addition of calcium whilst *KRT10* and *IVL*, (**B–C**) markers of differentiation expressed in the suprabasal layer and granular layer respectively were increased by calcium. P-values less than 0.05 (**) or less than (*) by Student's t-test are shown. Expression of all three genes were normalised to the housekeeping gene GAPDH. **D**) Immune blotting for protein levels for the suprabasal cytokeratin marker (*KRT10*) and the granular layer protein *IVL* shows that the addition of calcium upregulates the expression of both these markers of differentiation at 48 hrs after the switch to a high calcium concentration in comparison to the samples maintained at a low calcium concentration [0.6 mM]. GAPDH was used as a loading control.
(TIF)

Figure S2 **Analysis of VZV infection in keratinocytes by flow cytometry.** Primary keratinocytes were infected with an m.o.i. of 0.2 and either maintained in a low calcium or high calcium media or switched to a high calcium media 3 days p.i. Cells were harvested every 24 hrs, fixed and stained for VZV IE62/gE-FITC and analysed by flow cytometry **A**) Left panel, representative plot of a negative stain, VZV positive cells are seen in the lower right quadrant all plots are representative of samples at day 5 p.i. **B**) VZV staining (open histograms) shown relative to the unstained control (grey filled histogram) at day 5 p.i. Percentage of stained cells in all three conditions over the time course using (**C**) cell-associated virus and (**D**) cell-free VZV.
(TIF)

Figure S3 **Verification of transcriptome data.** **A**) Analysis of the transcriptome data set confirmed that good correlation was observed between replicates and conditions. Boxplot showing the distributions of reads per gene in each sample. Boxes range from the mean to the 1^st^ and 3^rd^ quartile, whiskers extend to 1.5× IQR and outliers are represented by circles. **B**) Heatmap illustrating the level of correlation between the lanes. Samples are denoted as in Figure 1G and numbers indicate the batch in which the sample was run. Hierarchical clustering was performed using Pearson's correlation coefficients on scale-normalised data. **C**) Principal component analysis of human reads for all samples. Projections are shown for components 1 and 2. Sample conditions are denoted by shape (KCV:triangles; KV:x's; KC: circles and K:+'s). Batches are denoted by colour (red: batch 1; green: batch 2; blue: batch 3). Considering the first 2 components, samples cluster primarily by batch, with the exception of the KC samples and KV2 and KV5 which all cluster tightly together regardless of batch. 1.2 mM calcium was added to primary keratinocytes and RNA harvested after 48 hrs. Ten genes were amplified by qPCR (**D**) in duplicate (all normalised to GAPDH) and the average fold difference and ± stdev calculated. The qPCR data (bottom panel) was compared to the data obtained from the RNA-seq experiment (top panel). Analysis showed good correlation between the two methods, confirming the lack of bias in the library construction.
(TIF)

Figure S4 **Effects of extracellular calcium on primary keratinocytes grown in monolayer culture.** From the transcriptome dataset, the fold change in the expression of several known markers of differentiation between undifferentiated and differentiated keratinocytes (KC/K) is shown and is consistent with epidermal differentiation. Genes are divided into either basal, suprabasal or granular depending upon their expression in the epidermis.
(TIF)

Figure S5 **VZV alters cytokeratin expression.** **A**) Analysis of changes in epidermal development genes (GO0008544) in VZV infected differentiated keratinocytes from transcriptome data (KCV/KC). Dotted line indicates regions of two fold or greater change. Points above dotted line denote genes increased by VZV infection of differentiated keratinocytes and vice-versa. Epidermal cytokeratins significantly altered by VZV infection, are shown in open symbols. EM imaging of (**B**) uninfected and (**C**) VZV infected keratinocytes show that cytokeratins bundles (white chevron) are present under both conditions, scale bar = 2 µm.
(TIF)

Figure S6 **VZV virions in primary keratinocytes.** Representative fields of electron microscope images of VZV infected keratinocytes. Cells were either maintained in a low calcium media (**A**) or switched 3 days p.i. as per our model (**D**). **B–C and E–F**) show a higher magnification of image (**A**) and (**D**) respectively. Inset (**C**) scale bar = 200 nm. VZV is highly cell associated in both conditions. Note the high virion production seen in differentiated keratinocytes.
(TIF)

Table S1 **Summary of reads mapping to the human and VZV genome.** The number of paired-end reads mapping to the human (hg19) and VZV (pOka) genomes for each lane alongside various QC metrics. Sample conditions are denoted with the following key: primary human keratinocytes (K); addition 3.3×10^4^ I.U of VZV (V), addition of >1 mM calcium to culture media (C) or both (CV). Batches are represented by a number. Infected samples KCV1–3 show a lower percentage of reads mapped to the human genome due to a large proportion of reads mapping to the VZV genome. Total number of mapped reads varies with batch due to an upgrade in the Illumina reagents for batch 3. Similarly, the mean base quality is higher for batch 3 although so too is the fraction of paired-end reads that map to identical positions on the human or VZV genomes. Estimated library sizes are independent of batch.
(XLSX)

Table S2 **Effect of VZV on cytokeratins.** Sample conditions are denoted with the following key: primary human keratinocytes (K); addition 3.3×10^4^ I.U of VZV (V), addition of calcium [1.2 mM] to culture media (C) or both (CV). Mean counts per million (CPM) values are shown for each of the cytokeratins in each condition alongside log_2_ fold changes and FDR corrected P-values for four comparisons (KC/K, KCV/KC, KV/K and KCV/KV).
(XLSX)

Table S3 **Effect of VZV on desmosomal genes.** Sample conditions are denoted with the following key: primary human keratinocytes (K); addition 3.3×10^4^ I.U of VZV (V), addition of calcium to culture media (C) or both (CV). Mean counts per million (CPM) values are shown for each of the desmosomal and hemidesmosomal genes in each condition alongside log_2_ fold changes and FDR corrected P-values for four comparisons (KC/K, KCV/KC, KV/K and KCV/KV). VZV has a significant effect on DSC1 and DSG1 in differentiated keratinocytes.
(XLSX)

Table S4 **Effect of VZV on serine protease genes.** Sample conditions are denoted with the following key: primary human keratinocytes (K); addition 3.3×10^4^ I.U of VZV (V), addition of calcium to culture media (C) or both (CV). Mean counts per million (CPM) values are shown for each of the desmosomal and hemidesmosomal genes in each condition alongside log_2_ fold changes and FDR corrected P-values for four comparisons (KC/K, KCV/KC, KV/K and KCV/KV).
(XLSX)

Table S5 **VZV ORFs significantly up- or downregulated by the addition of calcium.** Sample conditions are denoted with the following key: primary human keratinocytes (K); addition 3.3×10^4^ I.U of VZV (V), addition of calcium to culture media (C) or both (CV). ORFs are listed in order of significance from KCV/KV comparisons (A) excluding and (B) including sample KV1. Differential analysis was performed using edgeR on TMM-normalised viral read counts alone. RPKM values are provided to enable comparison of relative transcript abundance within samples. (**A**) Only ORF14 is significantly up-regulated (p_FDR_<0.01) in KCV/KV when excluding sample KV1. (**B**) Both ORF14 and ORF55 are significantly up-regulated (p_FDR_<0.01) when including sample KV1. Using a less stringent FDR cut-off (p_FDR_<0.05), ORF14, ORF55, ORF4 and ORF43 are significantly differentially expressed in both comparisons, ORF64 only in (**A**) and ORF57 only in (**B**).
(XLSX)

Text S1 **List of all primer sequences used in this manuscript.**
(DOCX)

Text S2 **List of all antibodies used in this manuscript.**
(DOCX)
