# Correction: RNA-seq Analysis of Host and Viral Gene Expression Highlights Interaction between Varicella Zoster Virus and Keratinocyte Differentiation

**DOI:** 10.1371/journal.ppat.1004313

**Published:** 2014-07-22

**Authors:** The PLOS Pathogens Staff

There is an error in Figure 8. An incorrect file was uploaded. The GAPDH and gE from the cell lysates now correspond to the supernatants used to test for the expression of KLKs.

The corrected version of Figure 8 can be seen here.

**Figure 8:**
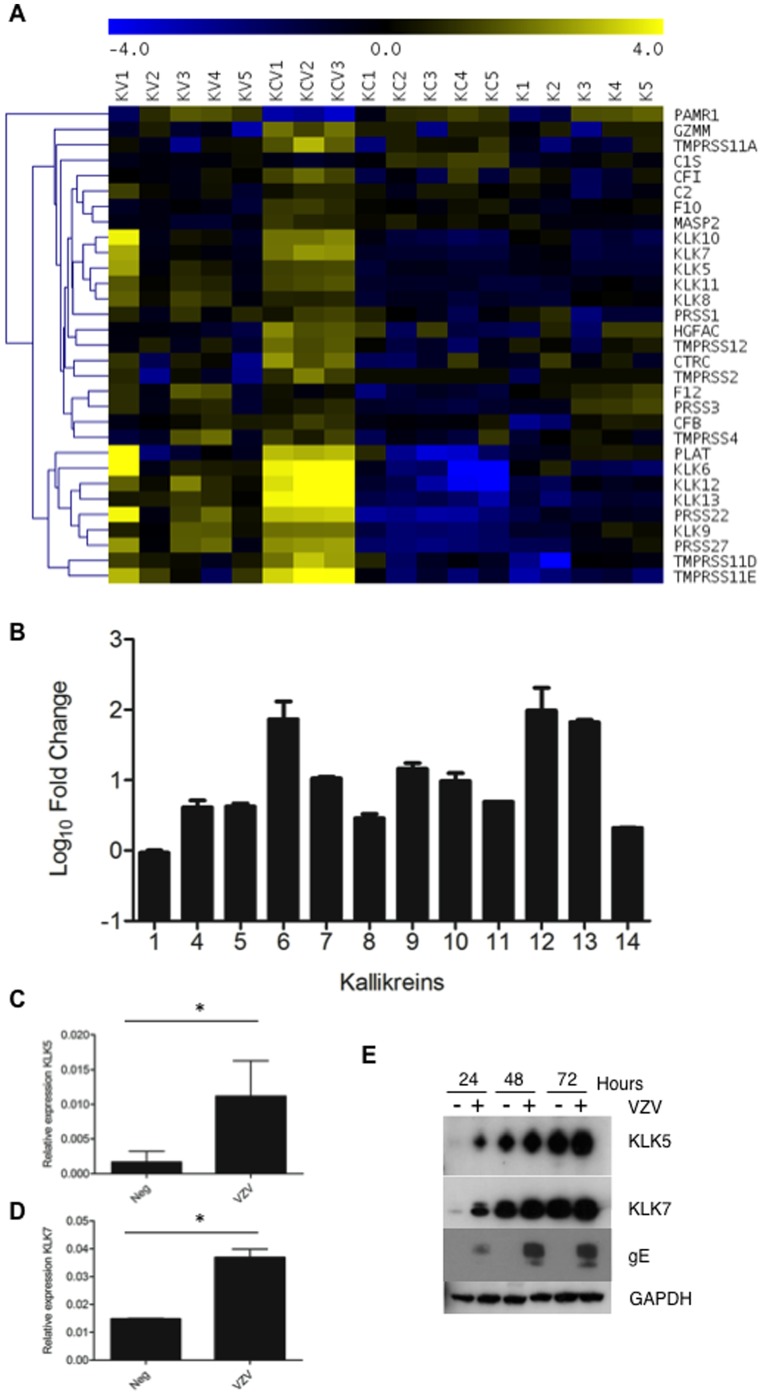
VZV infection increases KLK expression. A) Heatmap analysis of transcriptome changes in the serine peptidases and non-peptidase homologues group (IPR001314). A clear upregulation (yellow) of the majority of the genes in this group was observed in all KCV lanes. B) The majority of the kallikreins were upregulated in the differentiated keratinocytes compared to the uninfected cells (KCV/KC). Upregulation of KLKs in transcriptome of VZV infected differentiated keratinocytes was confirmed by qPCR for KLK5 and KLK7 (C–D). The relative expression of both genes ± standard deviation, normalised to GAPDH is shown for the uninfected and VZV infected samples at day 5 p.i. after the addition of calcium at day3, p values less than 0.05 are indicated (*). Immune blotting for KLK5 and 7 from concentrated supernatants of uninfected and VZV infected keratinocytes at 1–3 days post-differentiation (E). GAPDH and gE from the cell lysates was used as a loading control and to show VZV infection respectively.
