# Supplementary figures and images for: RNA-seq Analysis of Host and Viral Gene Expression Highlights Interaction between Varicella Zoster Virus and Keratinocyte Differentiation
Source: PLoS Pathog. 2014 Jan 30;10(1):e1003896. doi: 10.1371/journal.ppat.1003896 (PMC3907375; doi:10.1371/journal.ppat.1003896)

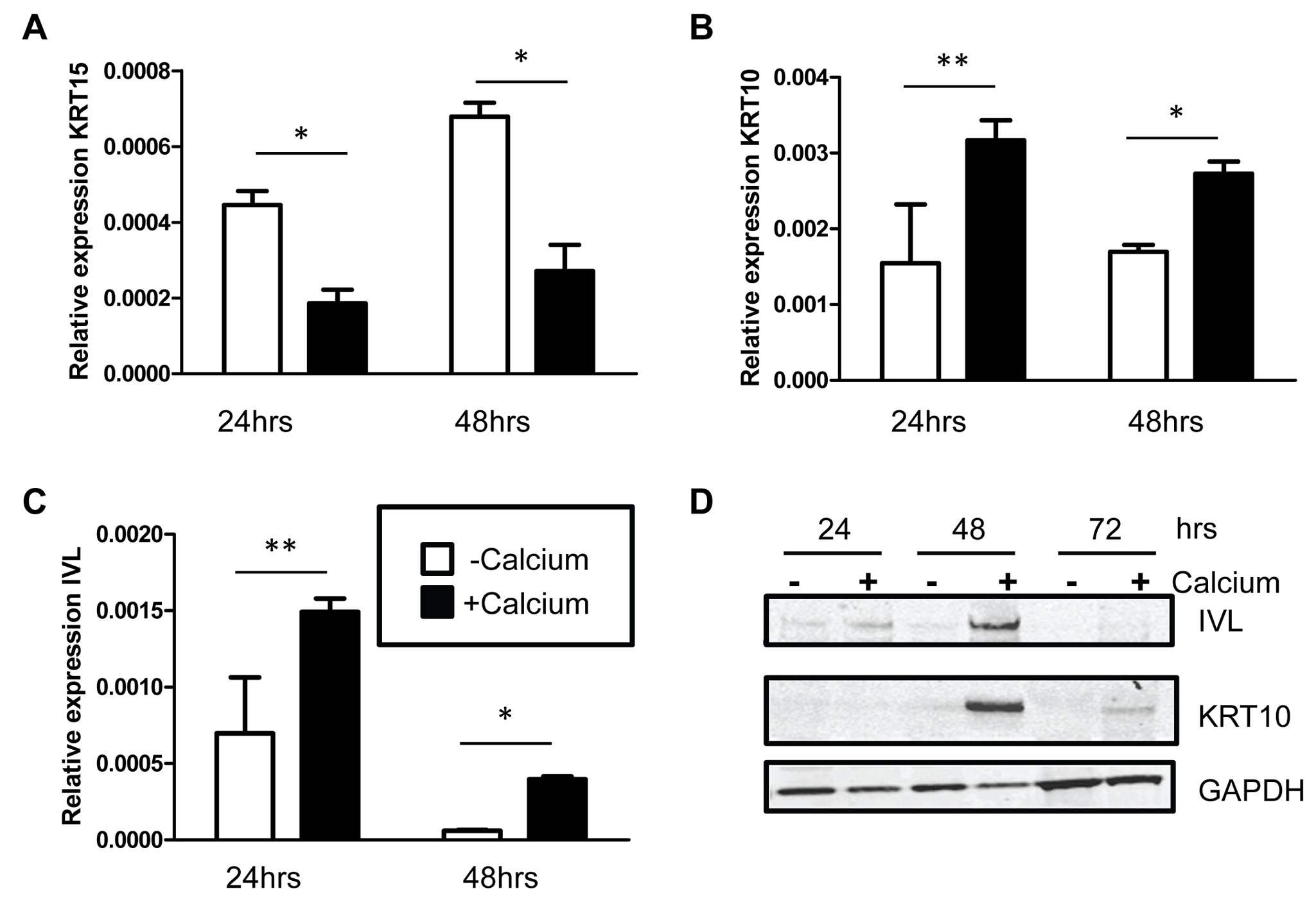

Supplement: Figure S1 — Increasing calcium concentration induces keratinocyte differentiation. Primary human keratinocytes grown either in low calcium [0.6 mM] or high calcium [1.2 mM] cultures were processed for total RNA and protein extraction. The increase of CaCl2 concentration to 1.2 mM initiated cellular differentiation of human keratinocytes beginning at 24 hrs, as detected by qPCR for (A) KRT15, a basal layer gene which was downregulated by the addition of calcium whilst KRT10 and IVL, (B–C) markers of differentiation expressed in the suprabasal layer and granular layer respectively were increased by calcium. P-values less than 0.05 (**) or less than (*) by Student's t-test are shown. Expression of all three genes were normalised to the housekeeping gene GAPDH. D) Immune blotting for protein levels for the suprabasal cytokeratin marker (KRT10) and the granular layer protein IVL shows that the addition of calcium upregulates the expression of both these markers of differentiation at 48 hrs after the switch to a high calcium concentration in comparison to the samples maintained at a low calcium concentration [0.6 mM]. GAPDH was used as a loading control. (TIF) [file ppat.1003896.s001.tif]

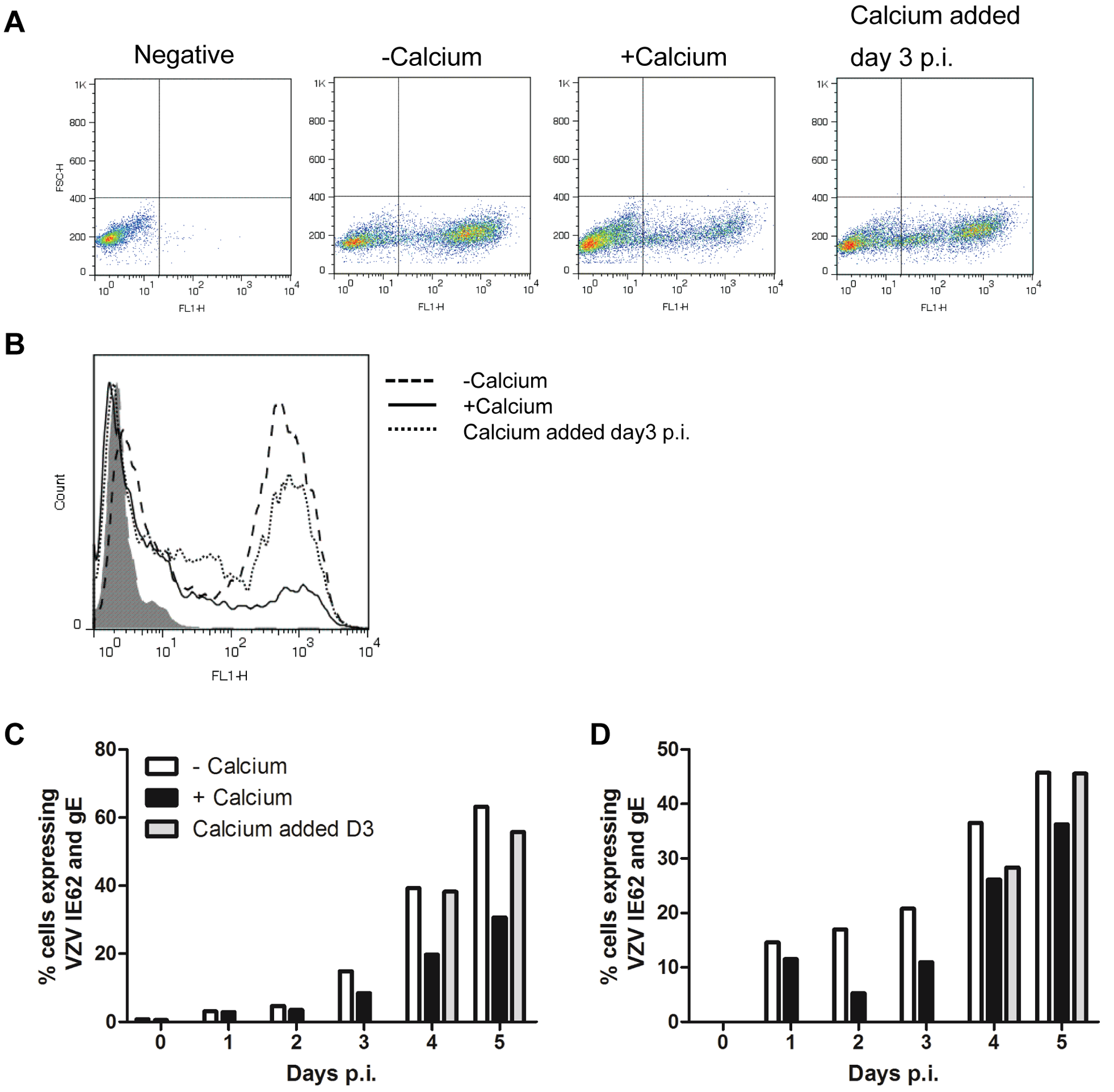

Supplement: Figure S2 — Analysis of VZV infection in keratinocytes by flow cytometry. Primary keratinocytes were infected with an m.o.i. of 0.2 and either maintained in a low calcium or high calcium media or switched to a high calcium media 3 days p.i. Cells were harvested every 24 hrs, fixed and stained for VZV IE62/gE-FITC and analysed by flow cytometry A) Left panel, representative plot of a negative stain, VZV positive cells are seen in the lower right quadrant all plots are representative of samples at day 5 p.i. B) VZV staining (open histograms) shown relative to the unstained control (grey filled histogram) at day 5 p.i. Percentage of stained cells in all three conditions over the time course using (C) cell-associated virus and (D) cell-free VZV. (TIF) [file ppat.1003896.s002.tif]

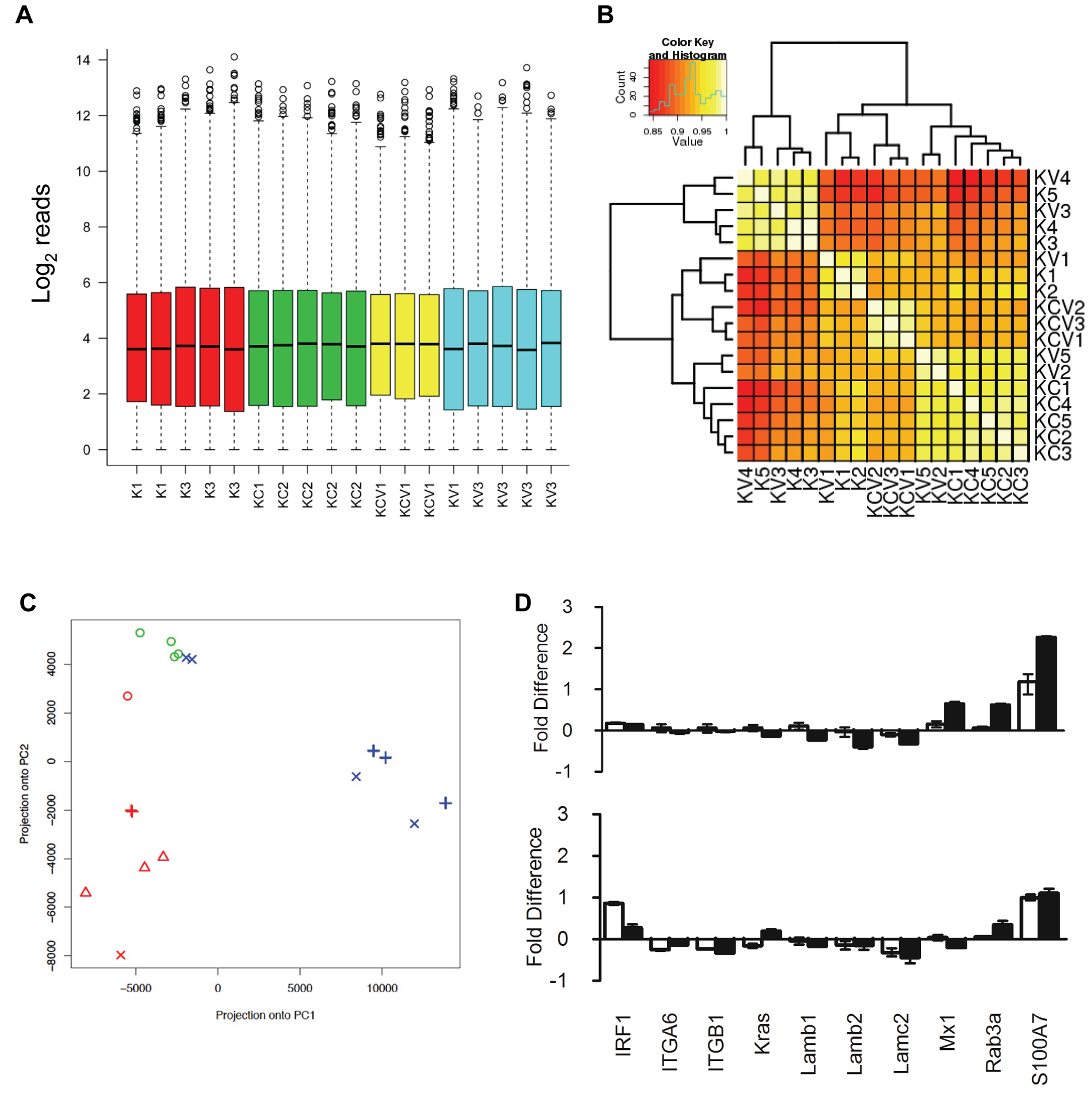

Supplement: Figure S3 — Verification of transcriptome data. A) Analysis of the transcriptome data set confirmed that good correlation was observed between replicates and conditions. Boxplot showing the distributions of reads per gene in each sample. Boxes range from the mean to the 1st and 3rd quartile, whiskers extend to 1.5× IQR and outliers are represented by circles. B) Heatmap illustrating the level of correlation between the lanes. Samples are denoted as in Figure 1G and numbers indicate the batch in which the sample was run. Hierarchical clustering was performed using Pearson's correlation coefficients on scale-normalised data. C) Principal component analysis of human reads for all samples. Projections are shown for components 1 and 2. Sample conditions are denoted by shape (KCV:triangles; KV:x's; KC: circles and K:+'s). Batches are denoted by colour (red: batch 1; green: batch 2; blue: batch 3). Considering the first 2 components, samples cluster primarily by batch, with the exception of the KC samples and KV2 and KV5 which all cluster tightly together regardless of batch. 1.2 mM calcium was added to primary keratinocytes and RNA harvested after 48 hrs. Ten genes were amplified by qPCR (D) in duplicate (all normalised to GAPDH) and the average fold difference and ± stdev calculated. The qPCR data (bottom panel) was compared to the data obtained from the RNA-seq experiment (top panel). Analysis showed good correlation between the two methods, confirming the lack of bias in the library construction. (TIF) [file ppat.1003896.s003.tif]

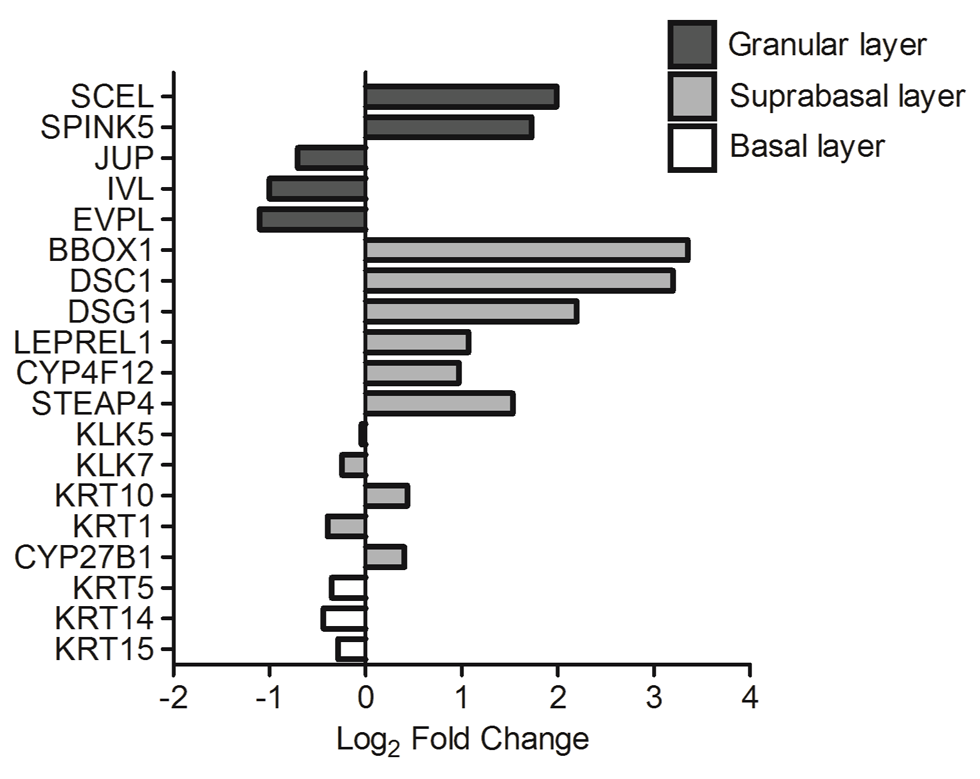

Supplement: Figure S4 — Effects of extracellular calcium on primary keratinocytes grown in monolayer culture. From the transcriptome dataset, the fold change in the expression of several known markers of differentiation between undifferentiated and differentiated keratinocytes (KC/K) is shown and is consistent with epidermal differentiation. Genes are divided into either basal, suprabasal or granular depending upon their expression in the epidermis. (TIF) [file ppat.1003896.s004.tif]

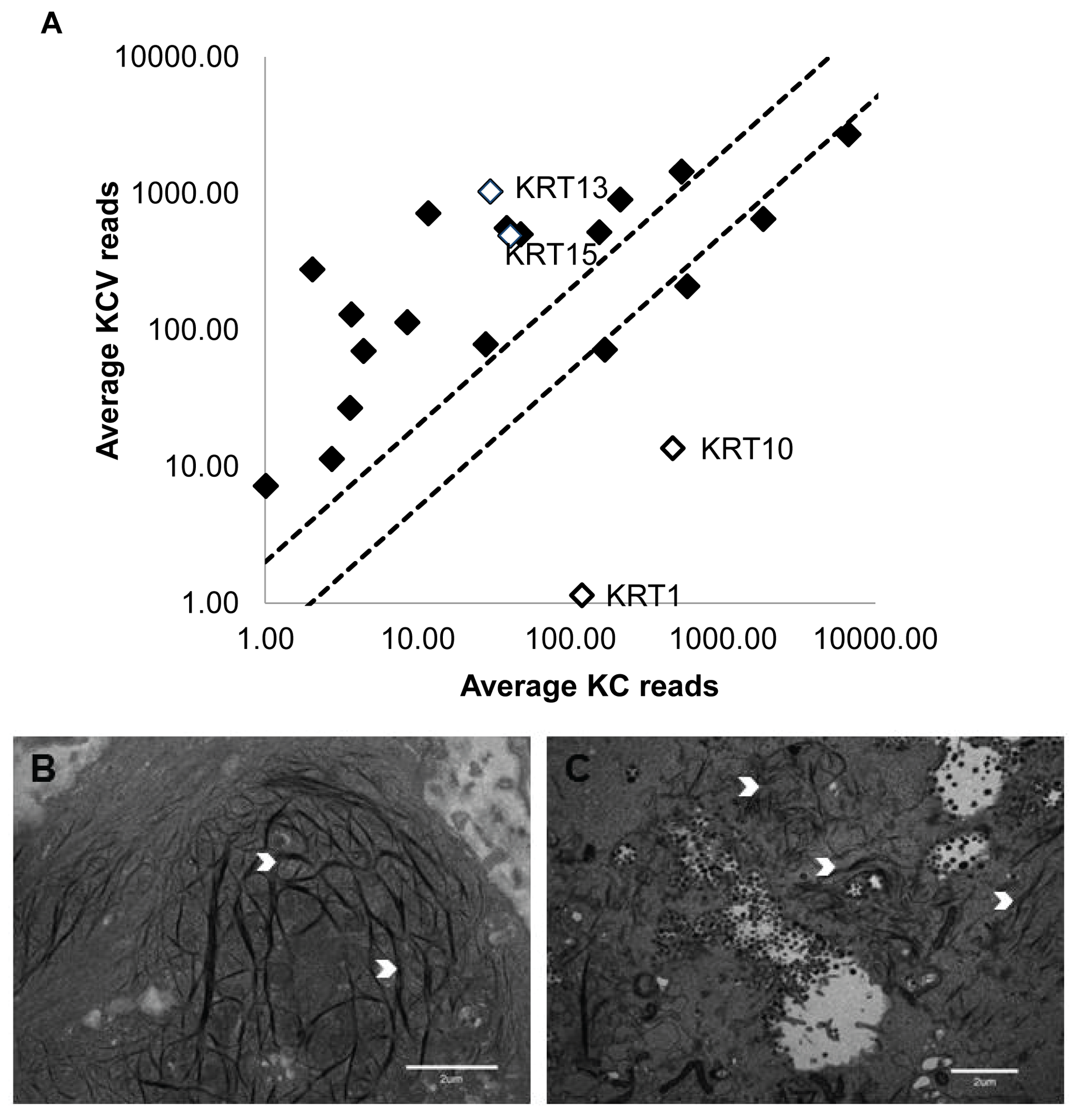

Supplement: Figure S5 — VZV alters cytokeratin expression. A) Analysis of changes in epidermal development genes (GO0008544) in VZV infected differentiated keratinocytes from transcriptome data (KCV/KC). Dotted line indicates regions of two fold or greater change. Points above dotted line denote genes increased by VZV infection of differentiated keratinocytes and vice-versa. Epidermal cytokeratins significantly altered by VZV infection, are shown in open symbols. EM imaging of (B) uninfected and (C) VZV infected keratinocytes show that cytokeratins bundles (white chevron) are present under both conditions, scale bar = 2 µm. (TIF) [file ppat.1003896.s005.tif]

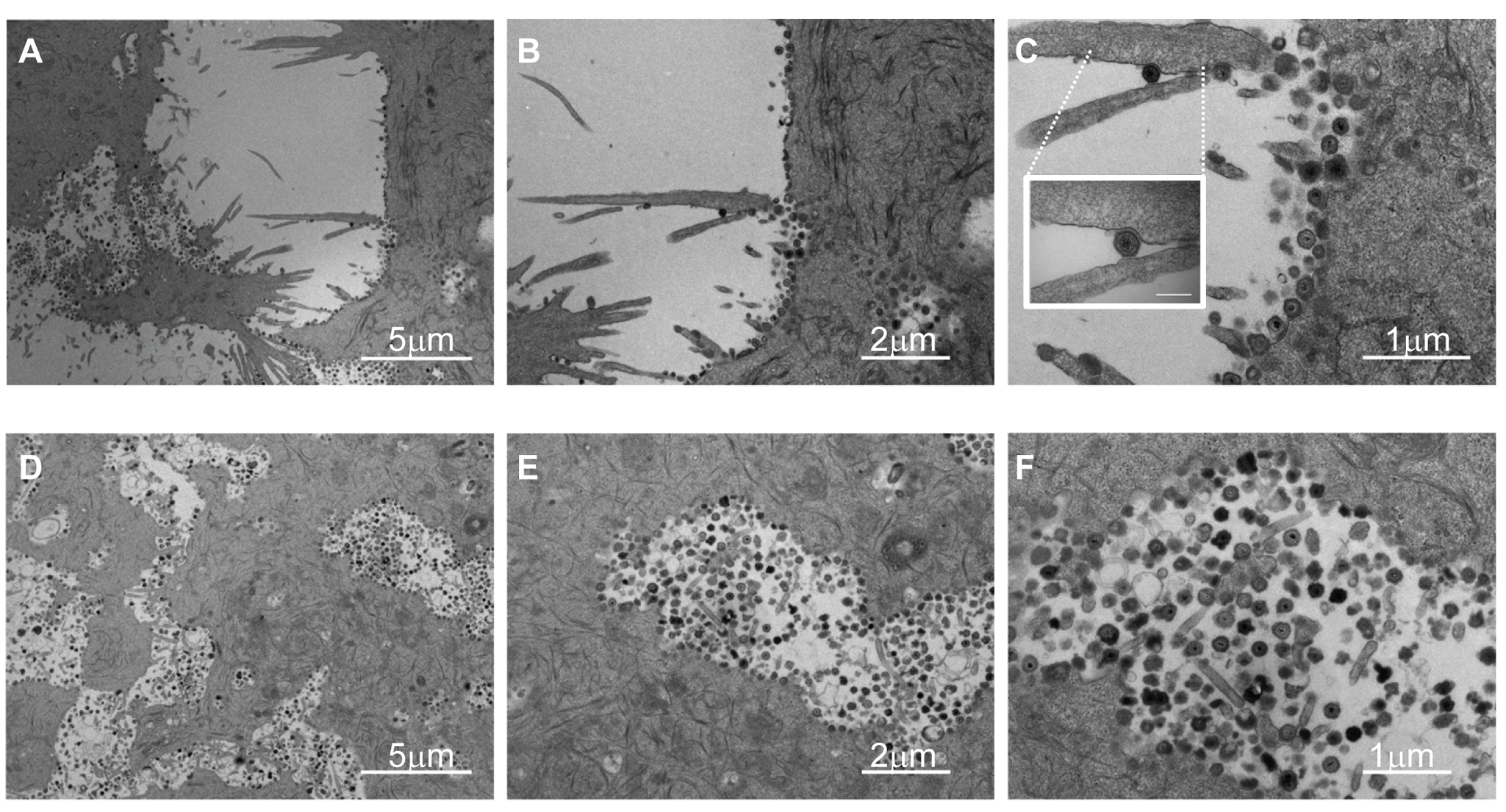

Supplement: Figure S6 — VZV virions in primary keratinocytes. Representative fields of electron microscope images of VZV infected keratinocytes. Cells were either maintained in a low calcium media (A) or switched 3 days p.i. as per our model (D). B–C and E–F) show a higher magnification of image (A) and (D) respectively. Inset (C) scale bar = 200 nm. VZV is highly cell associated in both conditions. Note the high virion production seen in differentiated keratinocytes. (TIF) [file ppat.1003896.s006.tif]
