# Supplementary material for: RNA-seq Analysis of Host and Viral Gene Expression Highlights Interaction between Varicella Zoster Virus and Keratinocyte Differentiation
Source: PLoS Pathog. 2014 Jan 30;10(1):e1003896. doi: 10.1371/journal.ppat.1003896 (PMC3907375; doi:10.1371/journal.ppat.1003896)
Supplement: Text S1 — List of all primer sequences used in this manuscript. (DOCX) [file ppat.1003896.s012.docx]

**Text S1**

| **Primer Name** | **Primer Sequence 5’-3’** |
| --- | --- |
| CYP26B1 F | CCGCTTCCATTACCTCCCGTTC |
| CYP26B1 R | CCACCGCCAGCACCTTCAG |
| DSC1 F | AGACTCCCCATGCAGACATC |
| DSC1 R | GTC CAC CTC CTT TGT TGG AA |
| DSC2 F | ACAGATGCCAAAACCAATGA |
| DSC2 R | CTGTGCTCATGGCTGATCTT |
| DSC3 F | ACTCAGTGTCGTGCGACTTC |
| DSC3 R | TTGCACCAAAAACTCCACAT |
| DSG1 F | CATTGGAGAAGACCTGGATG |
| DSG1 R | GCAAACTGGTTCCCAGTTCCAA |
| DSG2 F | CAGTAGCTTCCCAGTTCCAA |
| DSG2 R | TCTAGAAGCCATTGGGTCAG |
| DSG3 F | TAAGCCTTGGTGTTGATGGT |
| DSG3 R | GCTCCTTGACTTCCTGACAA |
| GAPDH F | GAAGGTGAAGGTCGGAT |
| GAPDH R | CATGGGTGGAATCATATTGGA |
| ITGA6 F | ACCCAGATATTGCAGTTGGA |
| ITGA6 R | TTCGATCAAGGTCCATGTTT |
| ITGB1 F | GGATTCTCCAGAAGGTGGTT |
| ITGB1 R | GGTAAAACAATGCCACCAAG |
| IVL F | GGGTGGTTATTTATGTTTGGGTGG |
| IVL R | GCCAGGTCCAAGACATTCAAC |
| KLK5 F | ACGAACCTCTGCAAGTTCAC |
| KLK5 R | TCAACATCTCTGGGAAGGAA |
| KLK7 F | GTCGGAACTTGGATCACATC |
| KLK7 R | GCGCCATCAATAATCTTGTC |
| KRAS F | GCCTGCTGAAAATGACTGAATATAAAC |
| KRAS R | TGATTCTGAATTAGCTGTATCGTCAAG |
| KRT1 F | ATTTCTGAGCTGAATCGTGTGATC |
| KRT1 R | CTTGGCATCCTTGAGGGCATT |
| KRT4 F | AGATACCTTGGGCAATGACA |
| KRT4 R | CTTGTTCAGGTAGGCAGCAT |
| KRT5 F | GGCATCACCGTTCCTGGGTAACAG |
| KRT5 R | CCGCTCCGGAAGGACACACTT |
| KRT10 F | TGATGTGAATGTGGAAATGAATGC |
| KRT10 R | GTAGTCAGTTCCTTGCTCTTTTCA |
| KRT13 F | GCTTTGTTGACTTTGGTGCT |
| KRT13 R | CAGTCACGGATCTTCACCTC |
| KRT15 F | AGGGTCAGGAGGAGGATATG |
| KRT15 R | TTTTCTCATTGCCAGAGAGG |
| KRT19 F | CTTCCGAACCAAGTTTGAGA |
| KRT19 R | GCGTACTGATTTCCTCCTCA |
| LAMB1 F | GCATAGTGCTGCTGACATTG |
| LAMB1 R | GCTTCTTCCAGAGCTTCCTT |
| LAMB2 F | ACCAGCATCTTGACTTGCTC |
| LAMB2 R | GTTGCTCACAGGGCTAGGTA |
| LAMC2 F | AGTGGAAGGAGAGCTGGAAA |
| LAMC2 R | GATTGTAACCCCAGCGTTCT |
| MX1 F | AACAACCTGTGCAGCCAGTA |
| MX1 R | AAGGGCAACTCCTGAGAGTG |
| PI3 F | CCGCTGCTTGAAAGATACTG |
| PI3 R | GAATGGGAGGAGAATGGAC |
| RAB3A F | ACGACAAGAGGATCAAGCTG |
| RAB3A R | TAGGTCTTGATCTGGGTGGA |
| RN5S F | GATCTCGGAAGCTAAGCAGG |
| RB5S R | AAGCCTACAGCACCCGGTAT |
| S100A7 F | AGACGTGATGACAAGATTGAC |
| S100A7 R | TGTCCTTTTTCTCAAAGACGTC |
| VZV ORF14 F | TGGAAATGGCTCTTTAATGGA |
| VZV ORF14 R | CCTTGGGACATTGGGTCTTA |
| VZV ORF29 F | CACGTATTTTCAGTCCTCTTCAAGTG |
| VZV ORF29 R | TTAGACGTGGAGTTGACATCGTTT |
| VZV ORF63 F | TCTACGCCATCGGATGTAAT |
| VZV ORF63 R | AAGAATCGGTGCTCTCCTCT |
