# Supplementary material for: RNA-seq Analysis of Host and Viral Gene Expression Highlights Interaction between Varicella Zoster Virus and Keratinocyte Differentiation
Source: PLoS Pathog. 2014 Jan 30;10(1):e1003896. doi: 10.1371/journal.ppat.1003896 (PMC3907375; doi:10.1371/journal.ppat.1003896)
Supplement: Text S2 — List of all antibodies used in this manuscript. (DOCX) [file ppat.1003896.s013.docx]

**Text S2**

| **Antibody** | **MW KDa** | **Dilution** | **Company** | **Cat No.** |
| --- | --- | --- | --- | --- |
| DSC1 | 100 | WB 1:1000 | Abcam | Ab93206 |
| DSG1 | 114 | WB 1:500 | BD | 610273 |
| GAPDH | 36 | WB 1:5000 | Abcam | Ab9485 |
| IVL | 68 | WB 1:1000  IF 1:100 | Abcam | Ab17105 |
| KLK5 | 32 | WB 1:1000  IF 1:100 | Abcam | Ab28565 |
| KLK7 | 27 | WB 1:1000  IF 1:100 | Novus | AF2623 |
| KRT1 | 66 | WB 1: 1000  IF 1:100 | Novusbio | NB100-2756 |
| KRT5 | 58 | WB 1:110  IF 1:100 | Abcam | Ab76879 |
| KRT10 | 60 | WB 1:1000  IF 1:100 | Abcam | Ab76318 |
| KRT15 | 45 | WB 1:1000  IF 1:100 | Abcam | Ab52816 |
| VZV IE63 | 36 | WB 1:2500  IF 1:100 | Gift WR |  |
| VZV gE | 78 | IF 1:100 | Santa Cruz | Sc-56994 |
| Anti Goat HRP |  | 1:5000 | Dako | P0449 |
| Anti Mouse HRP |  | 1:10000 | Dako | P0447 |
| Anti Rabbit HRP |  | 1:5000 | Dako | P0448 |
| Alexa Fluor 568 anti mouse |  | 1:1000 | Life Technologies | A11004 |
| Alexa Fluor 568 anti rabbit |  | 1:1000 | Life Technologies | A11011 |
| Alexa Fluor 568 anti goat |  | 1:1000 | Life Technologies | A11079 |
| VZV mixed |  | 1:2000 | Meridian Life Sciences | C05108MA |
| Biotinylated anti-mouse IgG (H+L) |  | 1:1000 | Vector Labs | BA-9200 |
| Anti mouse alkaline phosphatase-conjugated streptavidin |  | 1:400 | Jackson ImmunoResearch | 016-050-084 |
